# The Importance of Metal‐Organic Framework Linker Atoms for CO_2_ Reduction: A DFT Study

**DOI:** 10.1002/advs.202522176

**Published:** 2026-04-28

**Authors:** Ugochukwu Nwosu, Samira Siahrostami

**Affiliations:** ^1^ Department of Chemistry Simon Fraser University Burnaby Canada

**Keywords:** CO_2_ reduction, DFT simulations, metal‐organic frameworks, single‐atom catalysts

## Abstract

Although the metal within the secondary building unit of a metal‐organic framework is frequently studied as an active site, there is significantly less work exploring the linker effects. This work employs density functional theory (DFT) calculations to evaluate how linker atoms influence the carbon dioxide reduction reaction (CO_2_RR) in a series of 2D copper‐based metal‐organic frameworks (Cu MOFs). Regarding CO_2_RR selectivity, our results suggest that linker and copper single atoms preferentially facilitate *COOH and *OCHO intermediate formation, respectively, and that linker atoms facilitate H‐shuttling to metal sites. Further, we find that the three Cu MOFs featuring NH‐coordinated copper atoms exhibit the highest activity as predicted by DFT calculations. Analysis of the atomic electronic structure reveals linker electrostatics as a descriptor for CO_2_ activation and proton adsorption.

## Graphical abstract

Using DFT, we examine the role of linker atoms in CO_2_ reduction on copper‐based metal organic frameworks (Cu MOFs). Our calculations reveal that linker atoms may serve as both CO_2_ and H‐shuttling sites and suggest linker electrostatics as a descriptor for linker activity.

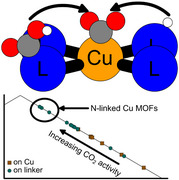

## 1 Introduction

Given the current imbalance in the global carbon cycle [1], it is imperative to derive solutions to rein in carbon emissions. The appeal of recycling carbon dioxide (CO_2_) into valuable chemicals manifests through two key advantages. Simultaneously, greenhouse gas emissions can be mitigated due to the conversion of CO_2_ and foregone by replacing existing carbon‐intensive processes with renewably powered alternatives. The electrochemical CO_2_ reduction reaction (CO_2_RR) stands out as a promising method for converting CO_2_ into valuable chemicals, offering a sustainable solution for chemical synthesis. However, the economic feasibility of CO_2_RR is hampered by the lack of active and selective catalysts.

Owing to its unique ability to yield C_2+_ products, copper features prominently in CO_2_RR catalysts [2]. However, metallic copper suffers from stability and selectivity issues [3, 4, 5, 6, 7]. To improve the CO_2_RR selectivity of copper and other metals, numerous catalyst paradigms have been employed, including oxide‐derived copper [8, 9, 10], doped carbon allotropes [11, 12, 13], single‐atom catalysts [14, 15, 16, 17, 18], and organic‐inorganic hybrids [10, 19, 20]. Among them, single‐metal‐atom‐doped carbon allotropes have garnered great promise in the reduction of CO_2_. For instance, nickel‐doped mesoporous carbon has shown 90% selectivity toward CO [21, 22]. The unique coordination chemistry of single metal atoms trapped in carbon provides vast opportunities for tuning the selectivity and activity of carbon‐based materials. However, it has been demonstrated that precisely controlling the coordination environment of a single metal atom is extremely challenging [14, 18]. Co‐doping nitrogen and metal atoms into carbon catalysts, for example, may result in a range of metal coordinations with a concomitant spread of activity and selectivity [14, 18]. On the other hand, maximum turnover frequencies and conversion rates require increasing the number of active sites. This calls for alternative materials with well‐defined coordination environments and a high density of active sites.

Due to their modularity, tunable active sites, and electrical conductivity [23, 24, 25, 26], metal‐organic frameworks (MOFs) are a promising platform for precise modification of metal coordination environment to improve selectivity for a single CO_2_RR product. In contrast to copper‐based MOFs (Cu MOFs) with cluster‐based secondary building units (SBUs) [27, 28, 29, 30, 31, 32, 33, 34, 35, 36, 37], which feature multiple metal atoms arranged in a cluster, Cu MOFs with node‐based SBUs feature a single metal atom coordinated by ligand linker atoms to form CuX*_n_* nodes (e.g., CuO_4_ [38, 39, 40, 41], Cu(NH)_4_ [42, 43, 44], CuS_4_ [45, 46, 47], and CuSe_4_ [48]). In principle, reactants can bind to every metal atom due to their isolation, making node‐based SBUs the upper limit of metal utilization in MOFs. Despite the immense design space, no suitable Cu MOF CO_2_RR catalysts exist, as Cu MOFs suffer from leaching and reconstruction issues and, importantly, do not yet achieve industrially feasible activity [49, 50, 51].

To predict activity from the properties of MOFs and related single‐atom catalysts, theoretical studies correlate the *d*‐band center, coordination number, and linker electronegativity to the activity of the metal center [14, 16, 52, 53, 54, 55, 56, 57, 58]; however, we believe that the focus on the metal atom overlooks a crucial aspect of SBU design for Cu MOF catalysts: *the linker atoms*. The catalytic role of linker atoms in Cu MOFs is readily suggested by that of similar motifs in molecular catalysts (viz. ligand non‐innocence [59, 60, 61], π‐acidity [60, 62], and second coordination sphere tuning [63, 64, 65, 66, 67]), the redox activity of O‐ [68, 69], NH‐ [70], and S‐linked [46] node‐based SBUs, and previous studies of H‐shuttling on MOF and Cu metal surfaces [71, 72, 73, 74]. Ligand modification in Cu MOFs has been shown to improve CO_2_RR performance indirectly [35, 40, 41, 63, 75, 76, 77, 78, 79], and, circumstantially, node‐based Cu MOFs exhibiting >2e^–^ product formation commonly feature Cu(NH)_4_ nodes [19, 39, 43, 80, 81]. Furthermore, that metal‐linker separations in Cu MOFs are similar to optimal ranges for C─C coupling (2.78‐3.56 Å) [43] and Tafel elementary steps (≈2.8 Å for Pt(111)) [82, 83] implies that linker sites can facilitate C─C coupling and PCETs. These observations underscore the catalytic role of linker atoms in CO_2_RR, yet no computational or experimental studies systematically investigate this role. To the best of our knowledge, only a few studies on Cu MOFs consider linker atom active sites (Table S1). However, when linker atoms are treated as active sites, studies either propose interactions based on the proximity of intermediates to linkers in DFT‐optimized geometries [84, 85, 86] or report adsorption complexes with reactive intermediates on linker atoms [42, 86, 87, 88].

**SCHEME 1:**
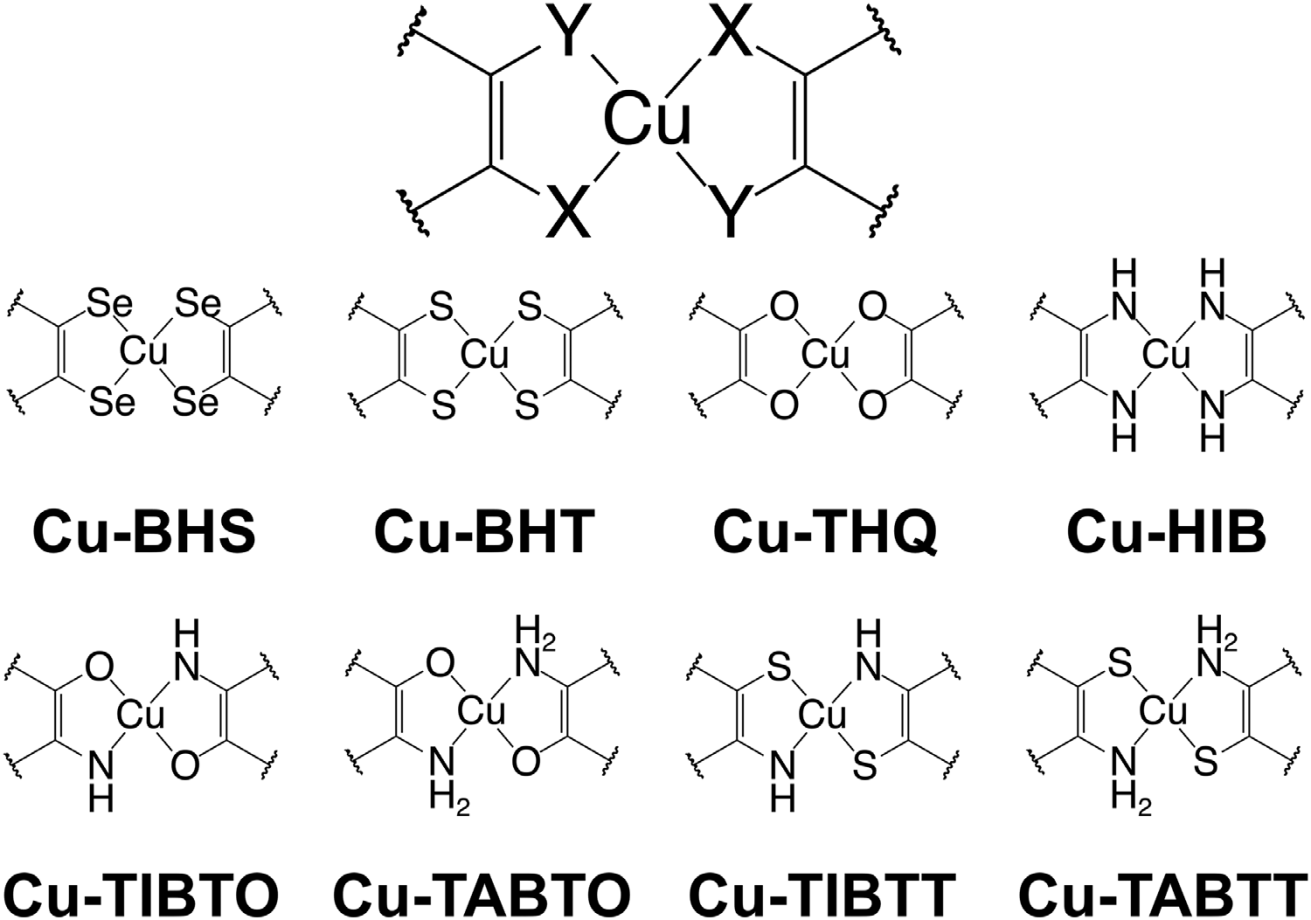
The secondary building units of eight Cu MOFs studied herein: copper tetrahydroquinone (Cu‐THQ), copper benzenehexathiol (Cu‐BHT), copper benzenehexaselenoate (Cu‐BHS), copper heximinobenzene (Cu‐HIB), copper triimino‐2,4,6‐benzenetriol (Cu‐TIBTO), copper triamino‐2,4,6‐benzenetriol (Cu‐TABTO), copper triimino‐2,4,6‐benzenetrithiol (Cu‐TIBTT), and copper triamino‐2,4,6‐benzenetrithiol (Cu‐TABTT).

In this study, we demonstrate the importance of linker atoms as catalytically active sites in Cu MOFs. Using DFT calculations we investigate CO_2_RR and HER for a collection of eight Cu MOFs featuring four‐coordinated node‐based SBUs (Scheme 1), copper tetrahydroquinone (Cu‐THQ), copper benzenehexathiol (Cu‐BHT), copper benzenehexaselenoate (Cu‐BHS), copper hexaiminobenzene (Cu‐HIB), copper triimino‐2,4,6‐benzenetriol (Cu‐TIBTO), copper triamino‐2,4,6‐benzenetriol (Cu‐TABTO), copper triimino‐2,4,6‐benzenetrithiol (Cu‐TIBTT), and copper triamino‐2,4,6‐benzenetrithiol (Cu‐TABTT) (for comments on the synthesizability of the Cu MOFs studied in this work, see Note S1). Our free energy calculations compare the reactivity of both linker and metal atoms and reveal site‐based selectivity originating from the net atomic charge: linker atoms are preferred sites of *COOH formation and adsorbed H, whereas *OCHO and *CO formation are preferred on copper atoms. We relate the site‐based selectivity through atomic charge analysis and present important considerations for kinetic models on Cu MOFs. This work represents the first systematic investigation of catalytic linker effects on Cu MOFs and supports the rational design of Cu MOFs to facilitate H‐shuttling and C─C coupling for >2e^–^ product formation.

## 2 Main Text

### 2.1 Cu MOF Structures

We performed unit‐cell optimizations using DFT‐calculated forces and strains to obtain equilibrium geometries for the Cu MOF catalysts (Figures S1–S8). Unit cell and SBU geometric parameters are listed in Tables S1 and S2, respectively. Cu‐BHS, Cu‐BHT, Cu‐TABTO, Cu‐TABTT, and Cu‐TIBTO exhibit hexagonal lattices with square planar copper node secondary building units (SBU dihedral angle: 0°). Cu‐HIB and Cu‐THQ also exhibit hexagonal lattices; however, the coordination geometry of the copper atom is between square planar and tetrahedral. The dihedral angles within the SBU of Cu‐HIB and THQ are 43° and 19°, respectively. The unit cell of Cu‐TIBTT is monoclinic–the copper atom being best described as tetrahedrally coordinated (SBU dihedral angle: 78°). We note that the structures of the previously synthesized Cu MOFs agree with the calculated lattice parameters (Table S2). Additionally, we find that although the average Cu─N distance is less than the average Cu─O distance, the average Cu–chalcogenide bond lengths increase with ionic radius (Cu─O < Cu─S < Cu─Se) and that Cu─L bond lengths can vary by up to 14%, depending on the identity of the other linker atom. For example, the Cu–N bonds in Cu‐TIBTO and Cu‐TABTO are 1.89 and 2.03 Å, respectively, while the Cu─O bonds in Cu‐TIBTO and Cu‐TABTO are 2.20 and 1.93 Å, respectively. As a final measure of structural stability, we have also performed projected crystal orbital Hamilton population (pCOHP) analysis (Figure S9). As pCOHPs are computed from the product of the density of states and Hamiltonian matrix elements, pCOHP plots provide insight into the bonding character of a material. In particular, the integrated pCOHP (IpCOHP) correlates with bonding strength; negative values of IpCOHP indicate stronger bonding. In general, we find that the IpCOHP for all Cu MOFs is negative, indicative of bonding character and the stability of the metal center.

**FIGURE 1:**
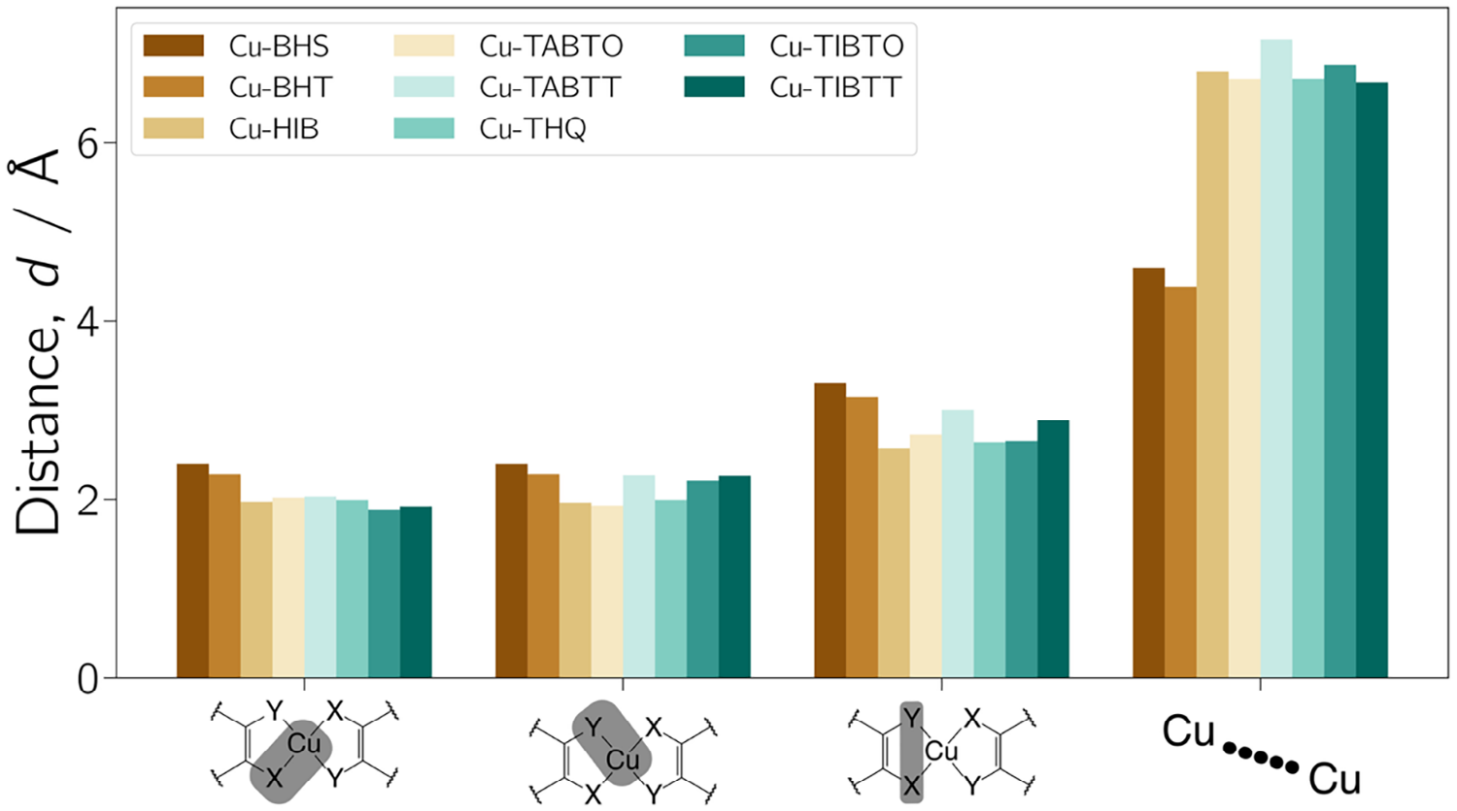
Atomic separations for linker and metal atoms in Cu‐MOF structures. From left to right, bar graph groups denote metal‐linker bond lengths for X and Y linkers, linker‐linker separations, and Cu─Cu separations between SBUs.

As the interplay of metal and linker sites is of interest, we have also analyzed the distances between adjacent adsorption sites (Figure 1 and Table S3). We note that for all Cu MOFs considered, the distances between metal‐metal sites are greater than 4.3 Å. In contrast, the maximum metal‐linker and linker‐linker separations are 2.4 and 3.3 Å, respectively. Direct C─C coupling mechanisms have been proposed for Cu MOFs; however, to the best of our knowledge, no such reports feature site‐site separations greater than 3.6 Å [50, 89, 90, 91, 92, 93]. Moreover, recent work highlights the role of surface‐adsorbed species as proton sources [74]. Inherently, surface‐mediated (or Langmuir‐Hinshelwood type) mechanisms are limited by the distance between adjacent sites. Thus, it follows that in Cu MOFs, the investigation of relevant site‐site interactions must consider linker involvement.

### 2.2 CO_2_RR Mechanisms on 2D Cu MOFs

To assess the CO_2_RR properties of each Cu MOF, we investigated the thermodynamics for the two‐electron reduction reactions forming CO and HCOO^–^. Following the mechanisms outlined in Equations (2)–(9), we determined the minimum energy paths (MEPs) for CO_2_RR. Note that each mechanism is preceded by an adsorption step (Equation 1), which may be accompanied by an electron transfer (Equation 2):

Mechanism 1:

(1)

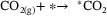



(2)

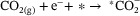




For each Cu MOF, the energies represent the minimum energy pathway (MEP) on the most stable site for each intermediate on the Cu MOF. The free energies and their components are tabulated in Tables S4–S10.

Mechanism 2:

(3)

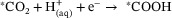



(4)

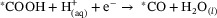



(5)

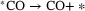

(6) $$\begin{array}{c} \mathrm{Total}:\;{\mathrm{CO}}_{2\left(g\right)}+2H_{\left(\mathrm{aq}\right)}^{+}+2e^{-}\to {\mathrm{CO}}_{\left(g\right)}+H_{2}O_{\left(l\right)}\;E^{0}=-0.10\;V \end{array}$$

Mechanism 3:

(7)

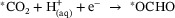



(8)

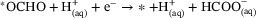

(9) $$\begin{array}{ccc} & & \mathrm{Total}:\;{\mathrm{CO}}_{2\left(g\right)}+2H_{\left(\mathrm{aq}\right)}^{+}+2e^{-}\to H_{\left(\mathrm{aq}\right)}^{+}+{{\mathrm{HCOO}}^{-}}_{\left(\mathrm{aq}\right)}\\ & & \;E^{0}=-0.12\;V \end{array}$$

**FIGURE 2:**
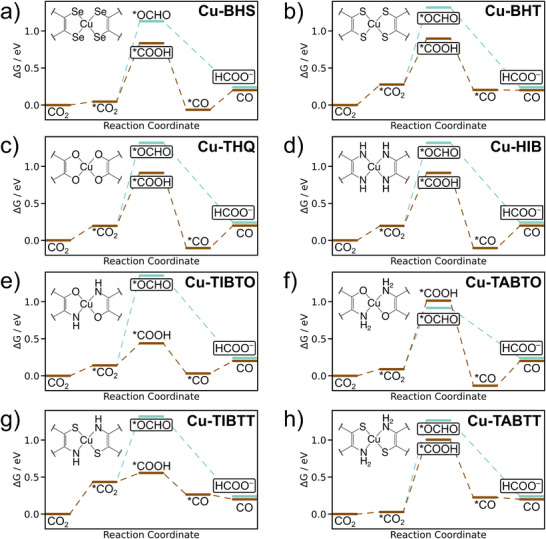
Free energy diagrams (at U = 0.0 V vs. RHE) for the MEP of CO_2_RR on the most stable sites of (a) Cu‐BHS, (b) Cu‐BHT, (c) Cu‐THQ, (d) Cu‐HIB, (e) Cu‐TIBTO, (f) Cu‐TABTO, (g) Cu‐TIBTT, and (h) Cu‐TABTT. The most stable sites are defined as the sites with the lowest adsorption free energy for a given intermediate.

Figure 2 displays the free energy diagrams for CO_2_RR for all Cu MOFs shown in Scheme 1, at U = 0.0 V vs. RHE. Product selectivity can be probed by following the MEP and comparing the relative adsorption free energies of key reaction intermediates for parallel mechanisms [2]. For Cu‐BHS (Figure 2a), Cu‐BHT (Figure 2b), Cu‐THQ (Figure 2c), Cu‐HIB (Figure 2d), Cu‐TIBTO (Figure 2e), Cu‐TIBTT (Figure 2g), and Cu‐TABTT (Figure 2h), the MEP for CO_2_RR results in *CO formation. For these seven Cu MOFs, the formation of *COOH occurs before that of *OCHO. CO binds moderately on Cu‐BHS, Cu‐THQ, Cu‐HIB, and Cu‐TIBTO (−0.33 eV < ΔG _(CO,ads)_ <−0.17 eV, Figure S10), indicating the possibility for CO reduction; CO binds weakly on Cu‐BHT, Cu‐TIBTT, and Cu‐TABTT (0.0 eV < ΔG_(CO, ads)_ < 0.09 eV, Figure S10), indicating CO as the CO_2_RR product. On Cu‐TABTO (Figure 2f), however, *OCHO formation is 0.1 eV less endergonic than *COOH formation, suggesting preferential HCOO^−^ formation. Interestingly, we note that CO is stabilized on S‐linked Cu MOFs over NH‐linked Cu MOFs as observed by Wu et al. [84]. For all Cu MOFs, the potential‐determining step (PDS) for 2e^–^ product formation is the first PCET (Equations 3 and 7 above) in accordance with previous work on Cu MOFs [10, 19, 27, 42, 43, 44, 79, 80, 81, 86, 87, 88, 90, 94, 95, 96]. Within the CHE framework, the applied potential introduces a uniform shift to all proton–electron transfer steps. Consequently, variations in the applied potential do not affect the relative energetics or mechanistic trends. Given that experimental CO_2_RR is typically performed at negative potentials, we additionally constructed the FEDs for the Cu MOFs at −1.0 V vs. RHE, a commonly employed experimental potential for CO_2_ reduction (Figure S11). We note that the aforementioned trends do not change when a potential of −1.0 V vs. RHE is applied.

Examining the trends in overpotentials (Figure S12), we observe that hydrogenation of the nitrogen atoms in the linker inhibits CO_2_RR on Cu MOFs. The NH_2_‐linked Cu MOFs, Cu‐TABTO and Cu‐TABTT, exhibit theoretical overpotentials of 0.83 and 0.98 V, respectively. These overpotentials represent 0.53 and 0.85 eV increases over their respective NH‐linked counterparts (Cu‐TIBTO and Cu‐TIBTT, respectively). In fact, of all catalysts, Cu‐TIBTT is predicted to have the lowest theoretical overpotential (0.12 V) for CO_2_RR, while Cu‐TABTT is predicted to have the highest overpotential (0.98 V). We suggest that this trend is caused by steric hindrance [78], whereby the additional hydrogens in the nitrogen linker “crowd out” the CO_2_RR intermediate. While this steric linker effect appears to be unique to N‐linked Cu MOFs, we will later show that linker atoms more generally influence the reaction thermodynamics across other Cu MOFs as well.

### 2.3 Selectivity Analysis

**FIGURE 3:**
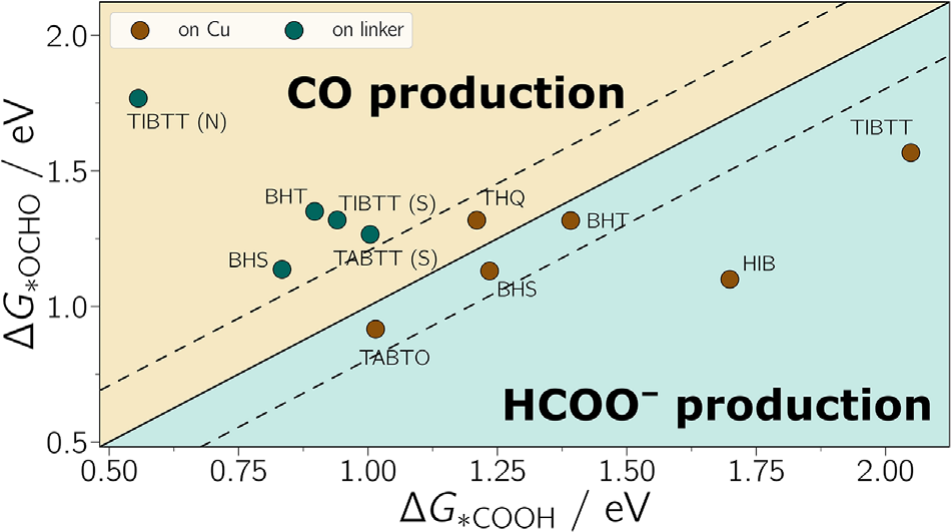
Selectivity plot for *OCHO vs. *COOH formation on Cu MOFs. Free energies are calculated at 0 V vs. RHE. The dashed lines are visual aids meant to indicate DFT error (0.2 eV). Note that only Cu MOF sites capable of forming bonded intermediates are included here. (see Note S3 for more details).

As shown in Figure 2, seven of the eight Cu MOFs studied are predicted to favor CO as the CO_2_RR product. To gain more insight into the nature of active site‐based CO vs. HCOO^–^ selectivity, we compared the adsorption free energies of *COOH and *OCHO (Figure 3). As *COOH and *OCHO formation have been identified as potential determining for CO and HCOO^−^, respectively, those sites for which $\Delta G_{*\mathrm{COOH}}$ exceeds $\Delta G_{*\mathrm{OCHO}}$, favor HCOO^−^. Conversely, those sites for which $\Delta G_{*\mathrm{OCHO}}$ exceeds $\Delta G_{*\mathrm{COOH}}$, favor CO. Sites for which $\Delta G_{*\mathrm{COOH}}\approx \Delta G_{\;*\mathrm{OCHO}}$ do not exhibit selectivity for either 2e^−^ product. Figure 3 shows that *COOH formation is favored over *OCHO formation on the linker atoms (by over 0.26 eV) while the opposite is generally true on Cu sites. This trend suggests that linker atoms are CO‐producing sites, while Cu sites are HCOO^−^‐producing sites. Cu‐THQ is an exception to this trend, as the Cu site is favored for *COOH. This is in agreement with experimental observations of CO selectivity of Cu‐THQ (Note S2) [88]. Further, while this contradicts previous reports comparing the formation of *COOH and *OCHO on Cu‐BHS and Cu‐BHT [94], we note that both Cu MOF sites lie within the dashed region, indicating a DFT error. At the extremes, we find that *COOH formation is favored by >0.9 eV on the Cu‐TIBTT N‐linker site and that *OCHO formation is favored by nearly 0.6 eV on the Cu‐HIB Cu‐site. That Cu‐TIBTT is expected to favor the formation of *COOH over that of *OCHO has been shown previously [87]. Given that most Cu MOFs are predicted to favor CO production, the site‐based selectivity of Cu MOFs suggests that the linker atoms of Cu MOFs play an active role in CO_2_RR.

However, our analysis of N‐linked Cu MOFs shows that hydrogenation of linker atoms inhibits CO_2_RR. Indeed, an additional challenge to selectivity lies in the hydrogen evolution reaction (HER, Equations (10), (11), (12)), as under reducing potentials, protons interact strongly with the electrode surface. Herein, we evaluate the energetics of the competing HER.
(10)

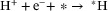



(11)

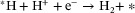

(12) $$\mathrm{Total}:\;2H^{+}+2e^{-}+*\to H_{2}\;\;E^{0}=0.0\;V$$

**FIGURE 4:**
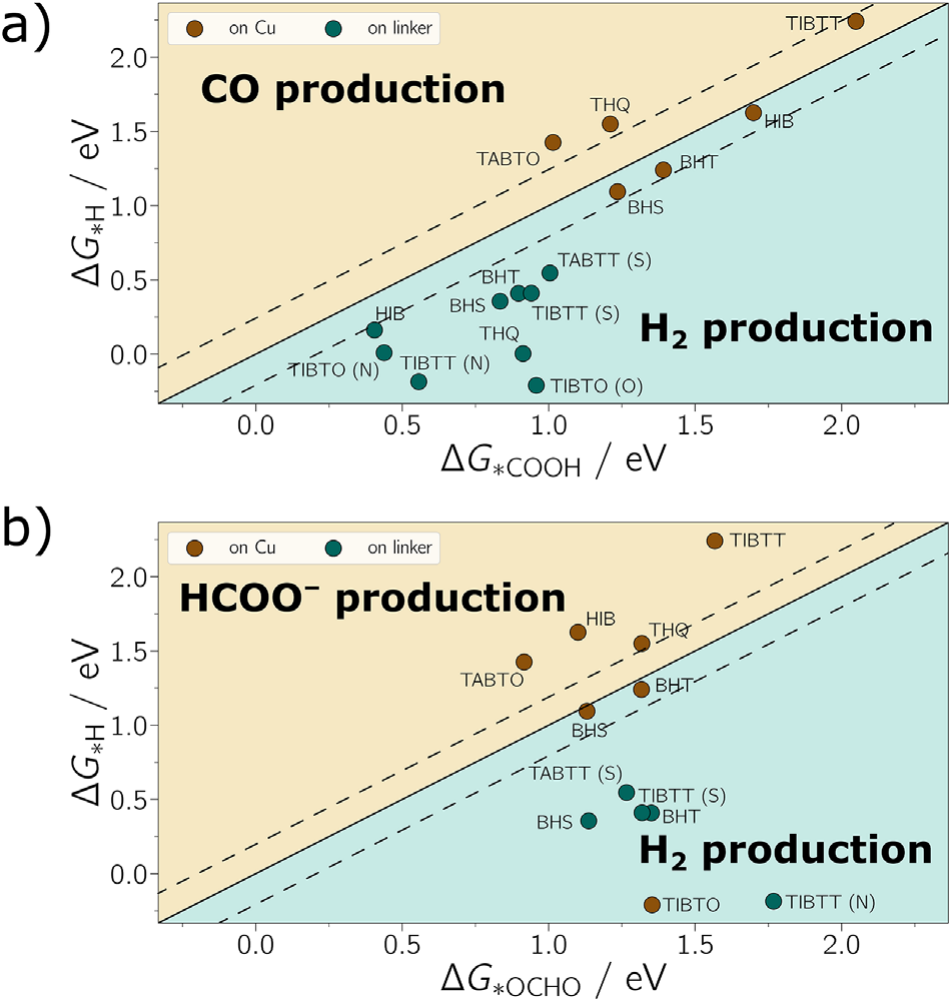
Selectivity plots for (a) *COOH vs. *H and (b) OCHO vs. *H on the metal and linker sites of Cu MOFs at 0 V vs. RHE.​ The dashed lines are visual aids meant to indicate DFT error (0.2 eV).

To probe CO_2_RR vs. HER selectivity, we compared the adsorption free energies of each CO_2_RR key intermediate to that of *H. Figure 4a compares $\Delta G_{\;*\mathrm{COOH}}$ to $\Delta G_{\;*H}$ for each Cu MOF on both the copper and linker atom sites. We observe that linker atoms favor *H formation over *COOH formation by more than 0.24 eV for all Cu MOFs studied. On Cu sites, however, *COOH formation is favored by Cu‐THQ and Cu‐TABTO by 0.34 and 0.41 eV, respectively, while *H formation is favored by less than 0.15 eV on Cu‐BHS, Cu‐BHT, Cu‐HIB, and Cu‐TIBTT. Figure 4b compares $\Delta G_{\;*\mathrm{OCHO}}$ to $\Delta G_{\;*H}$ for each Cu MOF on both the copper and linker atom sites. Once again, all linker atom sites preferentially form *H over *OCHO formation (by more than 0.55 eV), whereas Cu sites display mixed selectivity. In particular, the Cu sites of Cu‐THQ, Cu‐HIB, Cu‐TABTO, and Cu‐TIBTT favor *OCHO formation over *H formation (by over 0.23 eV), whereas those of Cu‐BHS and Cu‐BHT marginally favor *H formation (by less than 0.08 eV).

Overall, we note that for a given Cu MOF, protons absorb more readily on the linker atoms than on the copper atom (Figure S13). For example, the $\Delta G_{\;*H}$ on the nitrogen atom of Cu‐HIB is 0.17 eV, while it is 1.66 eV on the copper atom. On Cu‐BHT, H adsorption is favored on the S atom than over the Cu atom by 0.88 eV, differing slightly from the 1.038 eV gap found by Huang et al. [46]. Overall, we suggest that this site‐based selectivity arises from the fact that the linker atoms (e.g., O, S, Se, N) have a more negative charge density than the copper atom, which promotes proton adsorption. Note that N linker atoms exhibit weak H adsorption, suggestive of mobile surface H over H poisoning. Together with the fact that the linker atoms can facilitate CO_2_RR, this result indicates that there is strong competition between CO_2_RR and HER on the linker atoms.

At negative potentials, the free energy differences between PCET steps in Figure S13 will decrease to predict appreciable H coverage on linker sites and comparable *COOH formation on Cu and linker sites under CO_2_RR conditions. Previous calculations have shown that H‐shuttling is the preferred mechanism for CO_2_RR PCETs on copper surfaces and that the transition state barriers of the surface‐mediated H‐shuttling mechanism mirror experimental trends [71, 72, 73, 74]. We note that Tang et al. have explored H‐shuttling PCETs on a 2D Fe‐based MOF, Fe‐HIB [97], as have we for a series of 2D S‐linked MOFs [76]. Given the proximity of linker atoms to copper sites (1.89–2.40 Å; Figure 1; Table S3), linker‐adsorbed protons should be able to couple to Cu‐adsorbed intermediates. Thus, linker atoms may both shuttle protons to metal‐adsorbed intermediates and generate CO. This suggests two paradigms for improving the CO_2_RR performance of Cu MOFs: 1) tune H adsorption on linker atoms to facilitate proton transfer to Cu sites, and 2) tune CO_2_ activation on linker atoms to promote C─C coupling. We further explore the activity trends in linker active sites in the following section.

### 2.4 Activity Analysis

**FIGURE 5:**
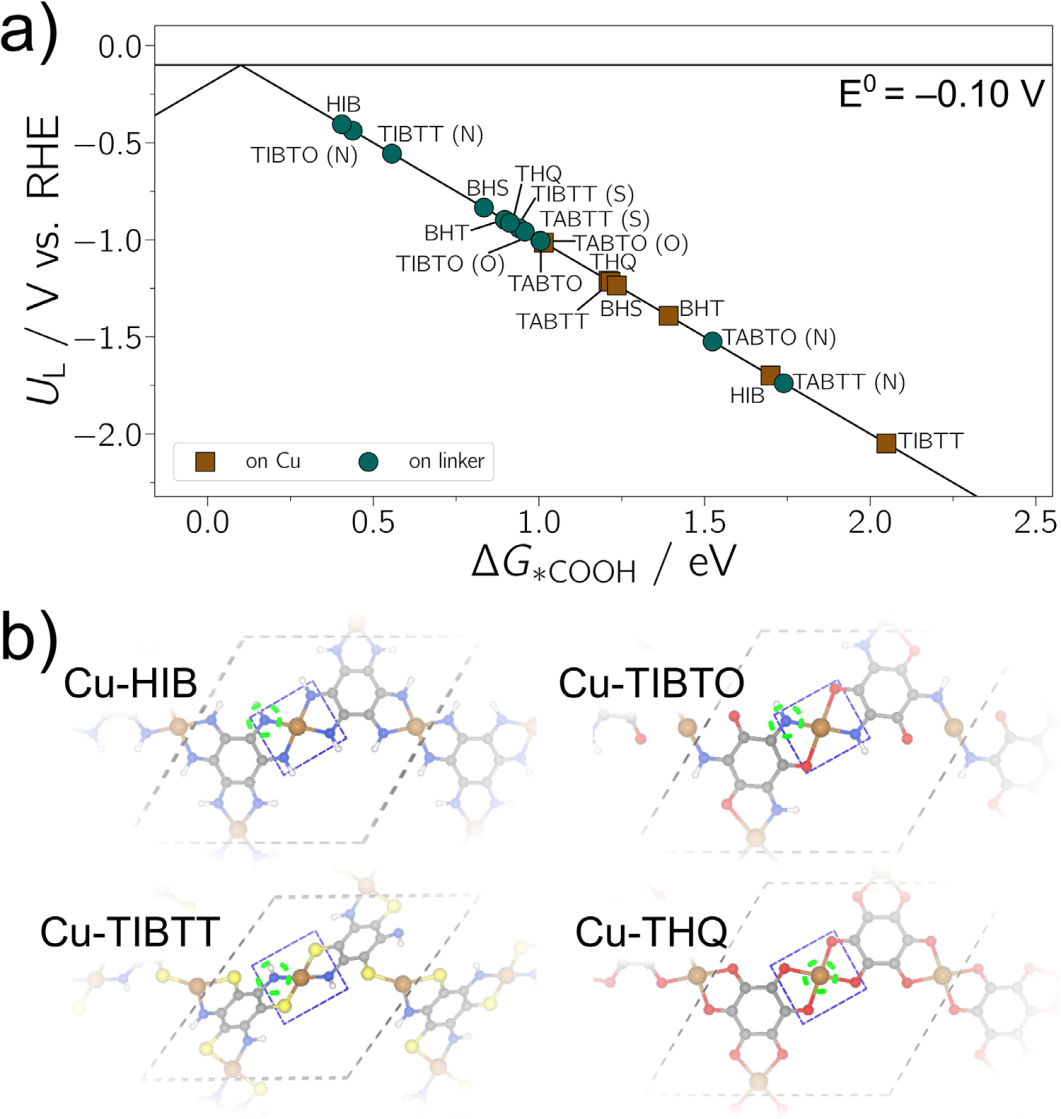
(a) Activity volcano for CO_2_RR production on Cu MOFs. Predicted activity correlates with proximity to the volcano's top. The horizontal line denotes the equilibrium potential for CO_2_ reduction to CO. (b) The atomic structures of the four selected Cu MOFs are shown, with active sites highlighted by green dashed circles. Cu‐HIB, Cu‐TIBTO, and Cu‐TIBTT are the most active Cu MOFs, all of which share the common feature that the linker nitrogen (N) serves as the active site. In contrast, Cu‐THQ active site is located on the copper (Cu) center.

Predicting CO_2_RR activity from thermodynamic calculations relies on assumptions of scaling relations and the Sabatier principle. BEP relations predict linear scaling relations between the reaction and activation free energies [98, 99, 100]. To identify CO_2_RR activity trends across various Cu MOFs, we constructed activity volcano plots for CO and HCOO^−^ (Figure 5; Figure S14). These plots relate the calculated limiting potentials to the free adsorption energies of *COOH and *OCHO, which were chosen as the activity descriptors. The calculated limiting potential (U_L_) is the potential at which all electrochemical reaction steps become thermodynamically downhill in the free energy diagram for each given product, i.e., CO or HCOO^−^ [101]. According to the Sabatier principle, the activity volcano can be divided into two regions: on the left side of the peak, overly strong binding of intermediates inhibits product formation, whereas on the right side, weak binding leads to reactant inactivation and reduced activity.

As shown in Figure 5 and Figure S14, all examined active sites lie on the right side of the activity volcano, suggesting that CO_2_ activation is the limiting process in these MOFs. Indeed, all Cu MOFs exhibit positive CO_2_ adsorption free energies (Table S6), and CO_2_ activation has been proposed as the rate‐determining step for CO_2_RR [102, 103]. The five most active sites are CO‐producing linker atom sites. Further, Figure 5a implies that the three Cu MOFs featuring NH linkers, Cu‐HIB, Cu‐TIBTO, and Cu‐TIBTO, are the most CO_2_RR active catalysts. While the two least active catalysts (Cu‐TABTO and Cu‐TABTT) also contain N‐based linkers, it is important to note that Cu‐TABTO and Cu‐TABTT incorporate NH_2_‐functionalized linkers, which, as we have discussed, tend to inhibit CO_2_RR activity. Moreover, the limiting potentials of the three Cu MOFs with NH linkers are 0.2 V lower than those of all other Cu MOFs. Thus, in agreement with previous studies of node‐based Cu MOFs [19, 39, 43, 80, 81], our calculations suggest that NH linkers are a key to CO_2_RR activity. We further explore the origin of this enhanced CO_2_RR activity by analyzing the atomic charges.

### 2.5 Linker Charge Analysis

Lastly, we sought to reconcile the observed effects of linker atoms on CO_2_RR reactivity with proposed electronic descriptors for single‐atom catalysts. By virtue of their structure, Cu MOFs present a theoretical challenge for identifying electronic effects on electrocatalytic activity. The periodic nature of Cu MOFs suggests that Cu MOFs should exhibit band‐like electronic structures like those observed in crystalline materials, such as transition metals and metal oxides. In such materials, the *d*‐band model, which relates the projected density of states (PDOS) to binding energies on metal surfaces, reliably predicts trends in metal binding energies [104, 105, 106, 107]. In contrast, the isolation of active sites in the SBU suggests that linker and metal atoms retain characteristics of their isolated electronic structures, akin to those of molecular catalysts. Ligand field theory thus describes ligation as a perturbation of the atomic electronic structure [108]. Alternatively, electrostatic interactions are universal: save for electron‐correlation effects, they are repulsive for like charges and attractive for opposites, regardless of the catalyst's periodic or single‐atom nature. Therefore, we examined the net atomic charges (NAC) on linker atoms to identify the origin of preferred adsorption on N linkers.

**FIGURE 6:**
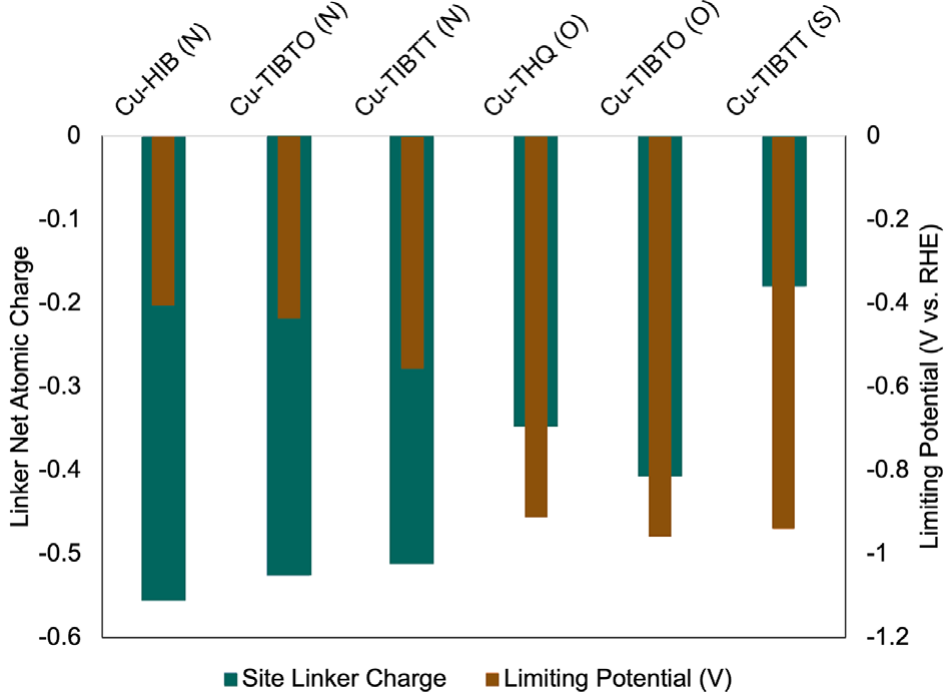
Net atomic charge on linker atom vs. limiting potential for CO production.

Figure 6 presents the NAC on the linker atom as well as the limiting potential for CO production on nitrogen and chalcogenide linker sites for a subset of Cu MOFs. This analysis shows that the sites with the lowest limiting potential (near the top of the volcano in Figure 5) feature the most negative linker net atomic charges (Table S11). The nitrogen linker atoms of Cu‐HIB, Cu‐TIBTO, and Cu‐TIBTT exhibit net atomic charges of −0.56 |e|, −0.53 |e|, and −0.50 |e|, respectively. Conversely, we find that those sites with the least negative net atomic charge exhibit the largest limiting potentials. The oxygen linker atoms of Cu‐THQ, Cu‐TIBTO, and Cu‐TIBTT exhibit net atomic charges of −0.34 |e|, −0.41 |e|, and −0.18 |e|, respectively. Although this contradicts what may be expected from the electronegativity of N and O atoms, we note that amines are known to be significantly more nucleophilic than their ether counterparts. As the proton is effectively a point charge, its potential near the surface is described by the electrostatic potential. For this reason, proton adsorption is generally favored on linker atoms. Further, as the values shown in Figure 6 are all negative, this indicates that the linker atoms uniformly exhibit more negative net atomic charge than the metal atom within the SBU, which is positively charged. This has previously been observed in Cu MOFs [42, 44]. We can now explain the site‐based selectivity observed for *COOH and *OCHO on Cu MOFs.

*OCHO and *COOH adsorption differ fundamentally in that surface binding occurs via O and C atoms, respectively [19, 39, 41, 42, 43, 44, 80, 81, 88, 94, 95]. The binding O and C atoms carry negative and positive charges in their respective adsorbates. It follows that *OCHO and *COOH exhibit divergent affinities depending on site polarity. Moreover, reports indicate that charge transfer to CO_2_ is required for CO_2_ activation on metallic surfaces [109, 110] and on 2D MOFs [87]. *COOH formation has also been observed to scale 1:1 with *H adsorption on the metal site of 2D NH‐linked MOFs, M‐HIB [97]. Simply put, net atomic charge is a binary indicator for site‐based selectivity. Between linker and Cu atoms, *OCHO prefers to bind to Cu because of favorable interactions between the positively charged metal and negatively charged O atoms; *COOH binds to linker atoms for the inverse reason. The trend for *COOH formation can be reframed, given that the above volcano analysis (Figure 5a) suggests that CO_2_RR is activation‐limited. Then, the preferred adsorption on nitrogen linker sites originates from the ability to initiate the charge transfer required to activate CO_2_. This result underscores the importance of linker design in Cu MOFs, as the most negatively charged atoms in the SBU are the linker atoms.

To visualize regions of charge accumulation and depletion in the Cu MOF structure, we further examined charge redistribution through valence charge density difference (VCDD) isosurfaces (Figure S15). Figure S15 shows the VCDD isosurfaces for the four Cu MOFs examined: Cu‐TIBTO, Cu‐TIBTT, Cu‐HIB, and Cu‐THQ. The yellow regions, primarily localized around the linker atoms, highlight these atoms as favorable sites for H adsorption and reveal negative charge density between the linker atoms and the Cu centers. This charge distribution indicates that the linker atoms play a key role in facilitating H atom transfer to the Cu sites. Altogether, our findings suggest that linker electrostatics may serve as a proxy for predicting Cu MOF CO_2_RR activity and site‐based selectivity.

## 3 Conclusion

In this work, we have demonstrated the importance of linker‐atom reactivity, despite the focus on the reactivity of the copper atoms in Cu MOFs. In the eight selected Cu MOFs considered here, the linker atoms are the preferred sites for *COOH and *H formation, while *OCHO formation is favored on the metal sites; site polarity serves as a binary indicator of the site‐based selectivity of *COOH and *OCHO. We find that NH‐linked Cu MOFs, Cu‐HIB, Cu‐TIBTO, and Cu‐TIBTT, are predicted to be the most active for two‐electron CO_2_RR to CO, with the nitrogen atoms of the linker serving as the preferred active site in each case. The participation of linker atoms in both HER and CO_2_RR mechanisms suggests a potential strategy for enhancing CO_2_RR on node‐based SBUs: tuning the electrostatic environment of linker atoms to facilitate proton shuttling and/or to promote the formation of adjacent reactive sites that enable C─C coupling. Finally, because site coverages appear explicitly in the rate expressions of microkinetic models, linker atoms cannot be treated purely as passive structural elements. In many existing models for CO_2_RR on node‐based MOFs or single‐atom catalysts, kinetic schemes focus primarily on metal‐centered pathways and implicitly assume that only metal sites host relevant intermediates. However, our findings indicate that linker atoms can stabilize key intermediates and may also host adsorbed species, such as hydrogen, that could participate in elementary reaction steps. As a result, the presence of reactive linker sites introduces the possibility of additional surface coverages and dual‐site reaction pathways that are not captured in simplified single‐site models. This observation suggests that more comprehensive kinetic descriptions, explicitly accounting for both metal and linker site balances and their associated intermediates, may be required to accurately describe CO_2_RR kinetics on copper‐based MOFs. Developing and validating such multi‐site microkinetic frameworks is an important direction for future work and lies beyond the scope of the present manuscript. Future work should expand upon this point and more explicitly consider pH‐ and potential‐dependent coverage effects.

## 4 Computational Section

### 4.1 Computational Details

Unless otherwise specified, DFT calculations were performed with the parameters specified herein. The Atomic Simulation Environment (ASE; version 3.23.1) [111] was used to perform DFT calculations with the Vienna Ab Initio Simulation Package (VASP; version 5.4.1) [112, 113, 114, 115]. Electronic wavefunctions were expanded in plane waves, and core electrons were treated using the VASP‐recommended projector‐augmented wave pseudopotentials (version 5.4) [116, 117]. A 450 eV plane‐wave kinetic energy cutoff and (4 × 4 × 1) Monkhorst‐Pack k‐point grid [118] were used. The electron exchange‐correlation was described by the generalized gradient approximation functional developed by Perdew, Burke, and Ernzerhof (PBE) [119]. For dispersion interactions, we applied Grimme's D3 correction scheme with Becke‐Johnson damping [120]. We applied Gaussian smearing to calculate the band occupancies with a smearing width of 0.04 eV. Spin‐polarization was considered for all calculations. Energies are converged to 10^−8^ eV within self‐consistent field cycles, and forces are minimized to below 0.01 eV/Å.

### 4.2 Unit Cell Optimization

We optimized the energy with respect to all ionic degrees of freedom (DOFs, atomic positions, cell volume, cell shape) using a method implemented in VASP (VASP keyword ISIF = 3). First, we generate starting structures based on powder X‐ray diffraction data and published structures (Table S2). When complete structural data were unavailable, starting atomic positions and unit cell parameters were extrapolated from similar structures. For example, because no unit cell data exists for Cu‐TABTT, the starting unit cell was assumed to be identical to that of Ni‐TABTT [121].

We then optimize the unit cell in three steps. We first allow all ionic DOFs to relax using an expanded basis set (900 eV energy cutoff). We then perform another geometry optimization, allowing only the atomic positions to relax (450 eV basis energy cutoff). Finally, we perform a single‐point energy calculation at the final geometry.

### 4.3 Adsorption Energy Calculations

For adsorption energy calculations, we constructed adsorption complexes by expanding the optimized unit cells such that the distance between periodic images in the direction normal to the catalyst surface was at least 20 Å. We explored a copper site and one of each linker type on each Cu MOF. For each site, the complex was optimized starting from adsorbate orientations parallel to and perpendicular to the surface. The lowest energy configuration is reported. We note that due to the shorter Cu─Cu distance observed in Cu‐BHS and Cu‐BHT, a bidentate adsorption mode is feasible for *OCHO. See Note S4 for a discussion of adsorption modes.

### 4.4 Free Energy Calculations

Gibbs, *G*, and Helmholtz, *A*, free energies for gas phase molecules and Cu MOF surfaces and complexes were calculated according to Equations (13), (14):

(13) $$G=E_{\mathrm{DFT}}+E_{\mathrm{ZPE}}+\int_{0}^{T}c_{P}dT-TS$$

(14) $$A=E_{\mathrm{DFT}}+E_{\mathrm{ZPE}}+\int_{0}^{T}c_{V}dT-TS$$

where *E*_DFT_ is the DFT‐calculated energy, *c*_P_ and *c_V_* are the heat capacities at constant pressure and volume, respectively, *T* is the temperature, *E*_ZPE_ is the zero‐point energy, *V* is the volume, and *S* is the entropy. Free energies for all species were calculated at 298 K and 1 bar. We calculated zero‐point energies and vibrational contributions to thermal and entropic terms from the second derivatives of the energy with respect to atomic positions using a finite‐difference method implemented in ASE (ase.vibrations.vibrations.Vibrations). For gas‐phase molecules, we calculate thermodynamic quantities using the ideal gas partition function (where we assume that the lowest 5 (6) frequencies correspond to translational and rotational degrees of freedom (DOFs) for linear (nonlinear) molecules); for adsorbate complexes, we consider only vibrational DOFs. All vibrational DOFs are treated harmonically, and catalyst DOFs were neglected. Further, we assume that the term is negligible on catalyst surfaces and in adsorbate complexes, so we have *A* ≈ *G*. Thermodynamic quantities are extracted from frequencies using the ase.thermochemistry.IdealGasThermo and ase.thermochemistry.HarmonicThermo classes in ASE.

### 4.5 Computational Hydrogen Electrode and Gibbs Free Energies

To calculate the free energies of the reactions depicted in Equations (3), (4), (5), (6), (7), (8), (9), (10), (11), (12)and account for electrode potential, we employed the computational hydrogen electrode (CHE) model introduced by Nørskov et al. [101]. That is, we assumed a linear correction to the free energy based on the electrode potential, given that the chemical potential of a proton‐electron pair is equal to that of ^1^/_2_ H_2(g)_ at 0 V vs. RHE_._ We then calculated the free energies of adsorption Δ*G*_ads_ via the equation:

(15) $$\Delta \;G_{ads}=\Delta E_{DFT}+\Delta E_{ZPE}+\int_{0}^{T}\Delta c\;dT-T\Delta S+neU$$

where *n* is the net number of electrons transferred between the reference state and the adsorbed state, *e* is the elementary charge, and *U* is the electrode potential. All differences in Equation (^15^) are computed according to stoichiometrically via Equations (3), (4), (5), (6), (7), (8), (9), (10), (11), (12).

Free energies for CO_2_RR intermediates were calculated relative to gas‐phase CO_2_, while the free energy of *H was calculated relative to gas‐phase H_2_.

### 4.6 Population Analysis

Net atomic charges were computed using DDEC6 population analysis as implemented in chargemol (2017‐09‐26 release) [122, 123, 124, 125, 126], as the DDEC6 method has been tested on metal‐organic frameworks and shown to outperform popular charge partitioning schemes such as DDEC3 and Bader charge analysis [122, 123, 124, 125, 126].

### 4.7 Projected Crystal Orbital Hamilton Population Analysis

Projected Crystal orbital Hamilton population (pCOHP) [127] analysis was performed to probe the bonding character surrounding the SBU. pCOHPs were calculated from −20 to 5 eV (relative to the Fermi level) using the Bunge basis set [128] with *s*, *p*, and *d* orbitals included for the projection. pCOHPs were calculated from the output of a single‐point calculation performed with a refined *k*‐point grid (16 × 16 × 1, Monkhorst‐Pack [118]). pCOHPs and integrated pCOHPs (IpCOHPs) were computed using the LOBSTER software (version 5.1.1) [129, 130, 131, 132, 133].

## Funding

Canada Research Chair award number CRC‐2022‐00280. Natural Sciences and Engineering Research Council (NSERC) of Canada (Discovery Grant No. RGPIN‐2023‐05298).

## Conflicts of Interest

The authors declare no conflict of interest.

## Supporting information




**Supporting File**: advs75042‐sup‐0001‐SuppMat.docx.

## Data Availability

The data that support the findings of this study are available on request from the corresponding author. The data are not publicly available due to privacy or ethical restrictions.
