# Supplementary material for: The Importance of Metal‐Organic Framework Linker Atoms for CO2 Reduction: A DFT Study
Source: Adv Sci (Weinh). 2026 Apr 28:e22176. Online ahead of print. doi: 10.1002/advs.202522176 (PMC13325935; doi:10.1002/advs.202522176)
Supplement: Supplementary file 1 — Supporting File: advs75042‐sup‐0001‐SuppMat.docx. [file ADVS-9999-e22176-s001.docx]

Supporting Information

The Importance of Metal-Organic Framework Linker Atoms for CO_2_ Reduction: A DFT Study

Ugochukwu Nwosu and Samira Siahrostami*

A. Ugochukwu Nwosu and Samira Siahrostami

Department of Chemistry, Simon Fraser University, Burnaby, Canada
E-mail: samira_siahrostami@sfu.ca

**Table of Contents**

List of Figures 2

List of Tables 4

Supplementary Note 1: Synthesizability of Cu MOFs 10

Supplementary Note 2: CO_2_RR on Cu-THQ 10

Structures of Cu MOFs 12

DFT-calculated Energies 19

Supplementary Note 3: Excluded Data Points for Cu MOFs in Figures 3 and 4 30

Supplementary Note 4: *OCHO on Cu-BHS and Cu-BHT 31

References 32

List of Figures

**Figure S1**. The unit cell of copper benzenehexaselenoate (Cu-BHS) a) top view and b) side view. The black dashed line denotes the unit cell. The blue dashed line denotes the CuX_4_ unit. 12

**Figure S2**. The unit cell of copper benzenehexathiol (Cu-BHT) a) top view and b) side view. The black dashed line denotes the unit cell. The blue dashed line denotes the CuX_4_ unit. 12

**Figure S3**. The unit cell of copper hexaiminobenzene (Cu-HIB) a) top view and b) side view. The black dashed line denotes the unit cell. The blue dashed line denotes the CuX4 unit. 13

**Figure S4**. The unit cell of copper 1,3,5-triamino-2,4,6-benzenetriol (Cu-TABTO) a) top view and b) side view. The black dashed line denotes the unit cell. The blue dashed line denotes the CuX_2_Y_2_ unit. 14

**Figure S5**. The unit cell of copper 1,3,5-triamino-2,4,6-benzenetrithiol (Cu-TABTT) a) top view and b) side view. The black dashed line denotes the unit cell. The blue dashed line denotes the CuX_2_Y_2_ unit. 14

**Figure S6**. The unit cell of Cu-THQ a) top view and b) side view. The black dashed line denotes the unit cell. The blue dashed line denotes the CuX_4_ unit. 15

**Figure S7**. The unit cell of copper 1,3,5-triimino-2,4,6-benzenetriol (Cu-TIBTO) a) top view and b) side view. The black dashed line denotes the unit cell. The blue dashed line denotes the CuX_2_Y_2_ unit. 15

**Figure S8**. The unit cell of copper 1,3,5-triimino-2,4,6-benzenetrithiol (Cu-TIBTT) a) top view and b) side view. The black dashed line denotes the unit cell. The blue dashed line denotes the CuX_2_Y_2_ unit. Note that the copper atom adopts more of a tetrahedral coordination geometry in contrast to the square planar structure exhibited by the seven other Cu MOFs. 16

**Figure S9**: Crystal orbital Hamilton population (COHP) analysis for a) Cu-BHS, b) Cu-BHT, c) Cu-HIB, d) Cu-TABTO, e) Cu-TABTT, f) Cu-THQ, g) Cu-TIBTO, and h) Cu-TIBTT. Energies are relative to the Fermi energy. COHPs are integrated up to the Fermi energy and displayed in each subfigure. 18

**Figure S10**. Gibbs free energies of adsorption for CO on Cu MOFs. 25

**Figure S11**: Free energy diagrams (at U = –1.0 V vs. RHE) for the MEP of CO_2_RR on the most stable sites of a) Cu-BHS, b) Cu-BHT, c) Cu-THQ, d) Cu-HIB, e) Cu-TIBTO, f) Cu-TABTO, g) Cu-TIBTT, and h) Cu-TABTT. The most stable sites are defined as the sites with the lowest adsorption free energy for a given intermediate. 26

**Figure S12**. Theoretical overpotentials for CO_2_RR for CO and HCOO^–^ formation on Cu MOFs. a) The overpotentials for CO and HCOO^–^ formation are plotted separately. b) Overpotentials for CO and HCOO^–^ are plotted as the difference $\eta CO2RR=\eta CO2RRCO-\eta CO2RRHCOO$. 27

**Figure S13**. Free energy diagrams (at U = 0.0 V vs. RHE) for HER on the Cu and linker sites of a) Cu-BHS, b) Cu-BHT, c) Cu-THQ, d) Cu-HIB, e) Cu-TIBTO, f) Cu-TABTO, g) Cu-TIBTT, and h) Cu-TABTT. 28

**Figure S14**. Activity volcano for HCOO^–^ production on Cu MOFs. 29

**Figure S15**. Valence charge density difference isosurfaces for a) Cu-HIB, b) Cu-TIBTO, c) Cu-TIBTT, and d) Cu-THQ. Isosurfaces are shown with an isovalue of 0.0005 Å^3^. Yellow and blue isosurfaces indicate regions of negative and positive charge accumulation, respectively. 30

List of Tables

**Table S1**: Summary of Cu MOF Linker CO_2_RR Reactivity 5

**Table S2**. DFT-calculated and experimental lattice parameters. 17

**Table S3**. Structural parameters for the SBU in Cu-MOF structures. 17

**Table S4**. DFT-calculated energies for clean Cu MOF surfaces. 19

**Table S5**. DFT-calculated energies for gas-phase molecules. 19

**Table S6**. DFT-calculated energies for *CO_2_ adsorption complexes. 19

**Table S7**. DFT-calculated energies for *COOH adsorption complexes. 20

**Table S8**. DFT-calculated energies for *OCHO adsorption complexes. 21

**Table S9**. DFT-calculated energies for *CO adsorption complexes. 22

**Table S10**. DFT-calculated energies for *H adsorption complexes. 23

**Table S11**: Linker net atomic charges. 29

Table S1: Summary of Cu MOF Linker CO_2_RR Reactivity

| Ref | Cu MOF | Secondary Building Unit | Adsorption sites/mechanism (experiment/computational) | Linker reactivity studied (Y/N?) | CO_2_RR products |
| --- | --- | --- | --- | --- | --- |
| ^[1]^ | CuAdeAce | Cu_2_(ade)_2_(ace)_2_ | No DFT | N | CH_3_OH, C_2_H_5_OH |
| ^[1]^ | HKUST-1 | Cu_2_(COO)_4_ | No DFT | N | CH_3_OH, C_2_H_5_OH |
| ^[2]^ | CuBi9 | Cu_2_(COO)_4_ | No DFT, Bi sites: *OCHO (and subsequent HCOO^–^) formation  Cu sites: alcohol formation, C–C coupling (LR type with free CH_3_OH and HCOO^–^), circumstantial evidence based on comparative performance of CuBiX MOFs | N | CO, HCOOH, CH_3_OH, C_2_H_4_, C_2_H_5_OH |
| ^[2]^ | CuBi12 | Cu_2_(COO)_4_ | No DFT, Bi sites: *OCHO (and subsequent HCOO^–^) formation  Cu sites: alcohol formation, C–C coupling (LR type with free CH_3_OH and HCOO^–^), circumstantial evidence based on comparative performance of CuBiX MOFs | N | CO, HCOOH, CH_3_OH, C_2_H_4_, C_2_H_5_OH |
| ^[2]^ | CuBi84 | Cu_2_(COO)_4_ | No DFT, Bi sites: *OCHO (and subsequent HCOO^–^) formation  Cu sites: alcohol formation, C–C coupling (LR type with free CH_3_OH and HCOO^–^), circumstantial evidence based on comparative performance of CuBiX MOFs | N | CO, HCOOH, CH_3_OH, C_2_H_4_, C_2_H_5_OH |
| ^[2]^ | Cu100 | Cu_2_(COO)_4_ | No DFT, Cu sites^†^ | N | CO, CH_3_OH, C_2_H_4_, C_2_H_5_OH |
| ^[3]^ | Cu-BDC | Cu_2_(COO)_4_ | No DFT, Cu metal, linker-assisted H_2_O activation^†^ | N | CO, HCOOH, CH_4_, C_2_H_4_, C_2_H_5_OH |
| ^[3]^ | NH_2_-Cu–BDC | Cu_2_(COO)_4_ | No DFT, Cu metal, linker-assisted H_2_O activation^†^ | N | CO, HCOOH, CH_4_, C_2_H_4_, C_2_H_5_OH |
| ^[3]^ | OH-Cu-BDC | Cu_2_(COO)_4_ | No DFT, Cu metal, linker-assisted H_2_O activation^†^ | N | CO, HCOOH, CH_4_, C_2_H_4_, C_2_H_5_OH |
| ^[3]^ | F-Cu-BDC | Cu_2_(COO)_4_ | No DFT, Cu metal, linker-assisted H_2_O activation^†^ | N | CO, HCOOH, CH_4_, C_2_H_4_, C_2_H_5_OH |
| ^[3]^ | 2F-Cu-BDC | Cu_2_(COO)_4_ | No DFT, Cu metal, linker-assisted H_2_O activation^†^ | N | CO, HCOOH, CH_4_, C_2_H_4_, C_2_H_5_OH, CH_3_COOH |
| ^[4]^ | NNU-50 | Cu_6_MePz | No DFT; *in situ* DRIFTs detection of *COOH, *CO, *CHO, *CH_2_O, *CH_3_O; Cu active site^†^ | N | CO, CH_4_, C_2_H_4_ |
| ^[5]^ | CR-MOF | CuN_2_S_2_ | No DFT; Cu active site^†^ | N | HCOOH |
| ^[6]^ | Cu_3_(HHTP)_2_ | CuO_4_ | *OCHO adsorbed on Cu with O (DFT); all on Cu | N | CH_3_OH |
| ^[6]^ | Cu_3_(HHTQ)_2_ | CuO_4_ | *OCHO adsorbed on Cu with O (DFT); all on Cu, PDS: *CO_2_ to *OCHO | N | CH_3_OH |
| ^[7]^ | HATNA-Cu-MOF | CuO_4_ | Ligand and CuO_4_ unit are redox active; Cu active site (change in Cu oxidation state after electrolysis);^†^ DFT only performed to compute π-π stacking energies | N | CO, CH_4_, C_2_H_4_ |
| ^[8]^ | Cu-DBC | CuO_5_ | C-bound *COOH (DFT) on Cu, O-bound *OCH_3_/*OCH_2_/*OH; cluster model without terminal H, PDS: *COOH -> *CO | N | CO, CH_4_, C_2_H_4_ |
| ^[9]^ | Cu-THQ | CuO_4_ | DFT: *COOH on O linker, *CO on Cu linker, *H adsorption on O linker, no comparison of adsorption energies on both sites, PDS: CO_2(g)_ -> *COOH, negative O Bader charge suggests linker H adsorption | Y | CO |
| ^[10]^ | CoPc-Cu-O | CuO_4_ | DFT: C-bound intermediates on metal active sites only for CO_2_RR and HER (Metropolis Monte Carlo with universal FF), examined concerted vs. sequential PCET (concerted preferred), PDS: CO_2(g)_ -> *COOH | Y | CO |
| ^[10]^ | CoPc-Cu-NH | CuN_4_ | DFT: C-bound intermediates on metal active sites only for CO_2_RR and HER (Metropolis Monte Carlo with universal FF), examined concerted vs. sequential PCET (concerted preferred) ), PDS: CO_2(g)_ -> *COOH | Y | CO |
| ^[11]^ | HKUST-1 | Cu_2_(COO)_4_ | No DFT, Undercoordinated Cu sites^†^ | N | CO, CH_4_, C_2_H_4_ |
| ^[11]^ | HKUST-1 (thermally treated) | Cu_2_(COO)_4_ | No DFT, Undercoordinated Cu sites^†^ | N | CO, CH_4_, C_2_H_4_ |
| ^[12]^ | PcCu-Cu-O | CuO_4_ | DFT: C-bound CO on metal active sites only for CO_2_RR; considers spillover C–C coupling via metal-site adsorption only | N | CO, CH_4_, C_2_H_4_ |
| ^[12]^ | PcCo-Cu-O | CuO_4_ | DFT: C-bound CO on metal active sites only for CO_2_RR; considers spillover C–C coupling via metal-site adsorption only | N | CO, CH_4_, C_2_H_4_ |
| ^[13]^ | HKUST-1 | Cu_2_(COO)_4_ | No DFT, suggests formation of a Cu–CO_2_ adduct based the mechanism of molecular CH_3_N/CO Cu complexes | N | oxalate |
| ^[14]^ | Cu_2_O@Cu-MOF | Cu_2_(COO)_4_ | “The surface Cu-MOF with strong adsorption ability to CO_2_ can tightly trap the CO_2_ molecules and enlarge the local CO_2_ concentration on the surface of the electrode. Also, it can further prevent Cu_2_O from the contact with the electrolyte to some degree, thus inhibiting the HER process. The rest of encapsulated Cu_2_O as a conducting medium is capable of accelerating charge transfer in comparison to that of Cu-MOF” ^[14]^; no DFT | N | CO, CH_4_, C_2_H_4_, HCOOH, C_2_H_5_OH |
| ^[15]^ | PCZ-2 | Cu_2_(COO)_4_ | No DFT, No explicit mechanistic insights; “synergistic effects” | N | CO |
| ^[16]^ | Cu-MOF-74 | Cu-O-C rod | DFT calculations performed but no information about active site provided | N | CO, HCOOH |
| ^[17]^ | CuO/Cu-MOF | Cu_2_(COO)_4_ | CuO enhances electron transfer | N | CO, HCOOH, CH_4_, C_2_H_4_ |
| ^[17]^ | Cu-MOF | Cu_2_(COO)_4_ | No DFT | N | CO, HCOOH, C_2_H_4_ |
| ^[17]^ | 2Cl-Cu-BDC | Cu_2_(COO)_4_ | No DFT, Cl inhibits CO_2_RR via steric effects^†^ | N | CO, HCOOH, CH_4_, C_2_H_4_ |
| ^[17]^ | 2Br-Cu-BDC | Cu_2_(COO)_4_ | No DFT, Br inhibits CO_2_RR via steric effects^†^ | N | CO, HCOOH, C_2_H_4_ |
| ^[17]^ | 2OH-Cu-BDC | Cu_2_(COO)_4_ | No DFT, OH inhibits CO_2_RR via steric effects^†^ | N | HCOOH, CH_4_ |
| ^[17]^ | 2NH_2_-Cu-BDC | Cu_2_(COO)_4_ | No DFT, NH_2_ promotes HER via H-donating amines^†^ | N | HCOOH, CH_4_, C_2_H_4_ |
| ^[17]^ | NH_2_-Cu-BDC | Cu_2_(COO)_4_ | No DFT, NH_2_ promotes HER via H-donating amines^†^ | N | HCOOH, CH_4_, C_2_H_4_ |
| ^[18]^ | PA-Cu-DBC-1 | CuO_5_ | DFT calculations performed on Cu atom within graphene defect, undercoordinated Cu active site assumed, C–bound intermediates | N | CO, CH_4_, C_2_H_4_ |
| ^[18]^ | PA-Cu-DBC-2 | CuO_5_ | DFT calculations performed on Cu atom within graphene defect, undercoordinated Cu active site assumed, C–bound intermediates | N | CO, CH_4_, C_2_H_4_ |
| ^[18]^ | Cu-DBC | CuO_5_ | DFT calculations performed on Cu atom within graphene defect, undercoordinated Cu active site assumed, C–bound intermediates | N | CO, CH_4_, C_2_H_4_ |
| ^[19]^ | Cu_2_(CuTCPP) | Cu_2_(COO)_4_ | No DFT | N | CO, HCOOH, CH_3_COOH |
| ^[20]^ | H-CuTCPP@Cu(OH)_2_ | Cu_2_(COO)_4_ | No DFT | N | HCOOH, CH_3_COOH |
| ^[20]^ | nH-CuTCPP@Cu(OH)_2_ | Cu_2_(COO)_4_ | No DFT | N | HCOOH, CH_3_COOH |
| ^[21]^ | Cu1 | CuN_2_ | DFT: O-bound *OCHO on Cu, PDS: CO_2(g)_ -> *OCHO, only Cu active site considered^†^ | N | CO |
| ^[21]^ | Cu2 | CuN_4_ | DFT: O-bound *OCHO on Cu, PDS: CO_2(g)_ -> *OCHO, only Cu active site considered^†^ | N | CO |
| ^[21]^ | Cu3 | CuN_4_ | DFT: O-bound *OCHO on Cu, PDS: CO_2(g)_ -> *OCHO, only Cu active site considered^†^ | N | CO |
| ^[22]^ | Cu_2_O@Cu-HHTP | CuO_4_ | Proposed that *COOH, *CO, *CHO, *CH_2_O, *OCH_3_ are adsorbed on Cu and interact with OH group of ligand; *operando* ATR-FTIR detects adsorbates but not ligand interactions; DFT model: HHTP ligand on Cu_2_O surface | N | CO, CH_4_, C_2_H_4_ |
| ^[22]^ | Cu-HHTP | CuO_4_ | No calculations performed or mechanism proposed | N | CO |
| ^[23]^ | Cu-DBC | CuO_5_ | DFT, H adsorption considered on O atoms, CO_2_RR assumed to occur on Cu, PDS: *CO -> *COH | Y | CO, CH_4_, C_2_H_4_ |
| ^[24]^ | Cu(111)@Cu-THQ | CuO_4_ | DFT: C-bound *COOH, *CO on Cu of Cu-THQ, PDS: CO_2(g)_ -> *COOH on Cu of Cu-THQ ; further reduction on Cu(111) surface | N | CO, CH_4_, C_2_H_4_ |
| ^[25]^ | Cu-HITP | CuN_4_ | DFT: C-bound intermediates on Cu active site only^†^; PDS: CO_2(g)_ -> *COOH | N | CO, HCOOH, CH_4_, CH_3_OH, C_2_H_4_, C_2_H_5_OH, CH_3_COOH |
| ^[25]^ | Cu-HITP@PDA | CuN_4_ | DFT (on Cu-HITP), Cu active site;^†^ polymer coating size intermediates and facilitate protonation | N | CO, HCOOH, CH_4_, CH_3_OH, C_2_H_4_, C_2_H_5_OH, CH_3_COOH, C_3_H_7_OH |
| ^[25]^ | Cu-HITP@PANI | CuN_4_ | DFT (on Cu-HITP), Cu active site;^†^ polymer coating stabilizes intermediates and facilitate protonation | N | CO, HCOOH, CH_4_, CH_3_OH, C_2_H_4_, C_2_H_5_OH, CH_3_COOH |
| ^[25]^ | Cu-HITP@PPV | CuN_4_ | DFT (on Cu-HITP), Cu active site;^†^ polymer coating stabilizes intermediates and facilitate protonation | N | CO, HCOOH, CH_4_, CH_3_OH, C_2_H_4_, C_2_H_5_OH, CH_3_COOH |
| ^[26]^ | PcCu-O_8_-Cu | CuO_4_ | DFT: C-bound on only metal sites considered^†^; PDS: CO_2(g)_ -> *COOH | N | CO |
| ^[26]^ | PcZn-O_8_-Cu | CuO_4_ | DFT: C-bound on only metal sites considered^†^; PDS: CO_2(g)_ -> *COOH | N | CO |
| ^[27]^ | CPF | Cu_2_(COO)_4_ | Assumed to be Cu atoms based on molecular porphyrin catalysts; no DFT | N | CO, HCOOH, CH_4_, C_2_H_4_ |
| ^[28]^ | CuBDC | Cu_2_(COO)_4_ | DFT used only to compute exfoliation energies; Cu active site^†^ | N | CO, HCOOH, CH_4_, C_2_H_4_, C_2_H_5_OH |
| ^[28]^ | Cu_2_BDC | Cu_2_(OH)_2_(COO)_4_ | DFT used only to compute exfoliation energies; Cu active site^†^ | N | CO, HCOOH, CH_4_, C_2_H_4_, C_2_H_5_OH |
| ^[29]^ | Cu_4_^II^-MFU-4l | Cu_4_Zn | Cu site^†^; aromatic H proposed to repel H_2_O and stabilize *CHO, DFT: PDS is *CHO -> *OCH_2_ | Y | CO, CH_4_ |
| ^[30]^ | MAF-2E | Cu_2_N_4_ | DFT: PDS: *CO -> *CHO (CH_4_ formation), *CO + *CO -> *CO-*CHO (C_2_H_4_ formation), Cu active site^†^ | N | CO, CH_4_, C_2_H_4_ |
| ^[30]^ | MAF-2ME | Cu_2_N_4_ | DFT, Cu active site^†^ | N | CO, CH_4_, C_2_H_4_ |
| ^[30]^ | MAF-2P | Cu_2_N_4_ | DFT, Cu active site^†^ | N | CO, CH_4_, C_2_H_4_ |
| ^[31]^ | Cu-BHT | CuS_4_ | DFT, H adsorption on Cu and S active sites | Y | H_2_ |
| ^[32]^ | HKUST-1 | Cu_2_(COO)_4_ | No DFT; Cu active site^†^ | N | n/a |
| ^[33]^ | NU2100 | N-coordinated Cu 4-cluster; Dihydrobenzo[1,2-d:4,5-d’]bis([1,2,3]triazole) linker | 100 Monte Carlo generated configurations; DFT: CO_2_, H_2_O, C_2_H_2_, C_2_H_4_, C_2_H_6_ found to strongly interact with N, H, C linker atoms, no catalysis considered | Y | n/a |
| ^[34]^ | Cu-TTB | chain consisting of N-linked tetrahedral Cu; 1,2,4,5-Tetra(1H-tetrazol-5-yl)benzene | DFT: Cu active sites^†^ | N | C_2_H_4_ |
| ^[35]^ | Cu-TEPT | Cu_3_C_3_ cluster; 2,4,6-tris(4-ethynylphenyl)-1,3,5-triazine | DFT: only Cu active sites^†^ | N | CH_4_ |
| ^[36]^ | CuTz-1-300 | 3,5-Diphenyl-1,2,4-triazole | CO_2_ is coordinated by counter anions, DFT | N | CH_3_OH |
| ^[37]^ | Cu-TIBTT | Cu(NH)_2_S_2_ | CO_2_ physisorbed over Cu atom, *COOH chemisorbed to C atom of ligand next to S atom, HCOOH physisorbed, CO physisorbed, no comparison of relative stability of active sites; the optimized geometries of the *COOH, *OCHO, and HCOOH, adsorption complexes other metal TIBTTs in the study exhibit binding over the C and S atoms in the ligand | Y | HCOOH, CO |
| ^[38]^ | Cu-THQ | CuO_4_ | DFT: C-bound *COOH, O-bound *OCHO; only Cu active sites considered^†^ | N | *OCHO |
| ^[38]^ | Cu-BHT | CuS_4_ | DFT: C-bound *COOH, O-bound *OCHO; only Cu active sites considered^†^ | N | *OCHO |
| ^[38]^ | Cu-BHS | CuSe_4_ | DFT: C-bound *COOH, O-bound *OCHO; only Cu active sites considered^†^ | N | *OCHO |
| ^[39]^ | Cu-HIB | Cu(NH)_4_ | DFT: C-bound *COOH^†^ | N | n/a |

^†^ No direct experimental/computational justification

Supplementary Note 1: Synthesizability of Cu MOFs

Cu-HIB has been experimentally synthesized and found to exhibit excellent stability in acidic and basic electrochemical conditions.^[40,41]^ Stable CO_2_RR performance has been demonstrated with Cu-THQ.^[9]^ And while Cu-TIBTO and Cu-TIBTT have not yet been experimentally synthesized, closely related Cu-TABTO (fully protonated Cu-TIBTO) has been synthesized and shown to exhibit stable electrical conductivity.^[42]^ Moreover, metal-substituted analogues such as Ni-TIBTO and Ni-TIBTT have been synthesized and tested under electrochemical conditions ^[42,43]^. Finally, the formation energy of Cu-TIBTT has also previously been computed to be –8.5 eV.^[37]^. Cu-THQ has been synthesized^[44]^ and tested for CO_2_RR.^[9]^ Negative formation energies have been computed for Cu-BHS and Cu-BHT,^[37,45]^ and both Cu MOFs have been synthesized. ^[46–48]^ Cu-BHT has been tested under electrochemical conditions.^[31,49]^

Supplementary Note 2: CO_2_RR on Cu-THQ

Regarding experimental studies of Cu-THQ: To the best of our knowledge, only 2 such studies exist.^[8,9]^ In the study by Majidi et al., the DFT calculations showed that *COOH formation was favoured over *OCHO formation by 0.3 eV. The DFT calculations performed by Liu et al. indicated that *COOH has 0.76 eV more stable than free CO_2_ and an open active site. However, we will note that Majidi et al. included implicit solvation, and Liu et al. modeled the CuO_4_ active site only (i.e., the benzene ligand was not included in their model). Both factors may contribute to the disparities in *COOH/*OCHO adsorption trends.

Structures of Cu MOFs


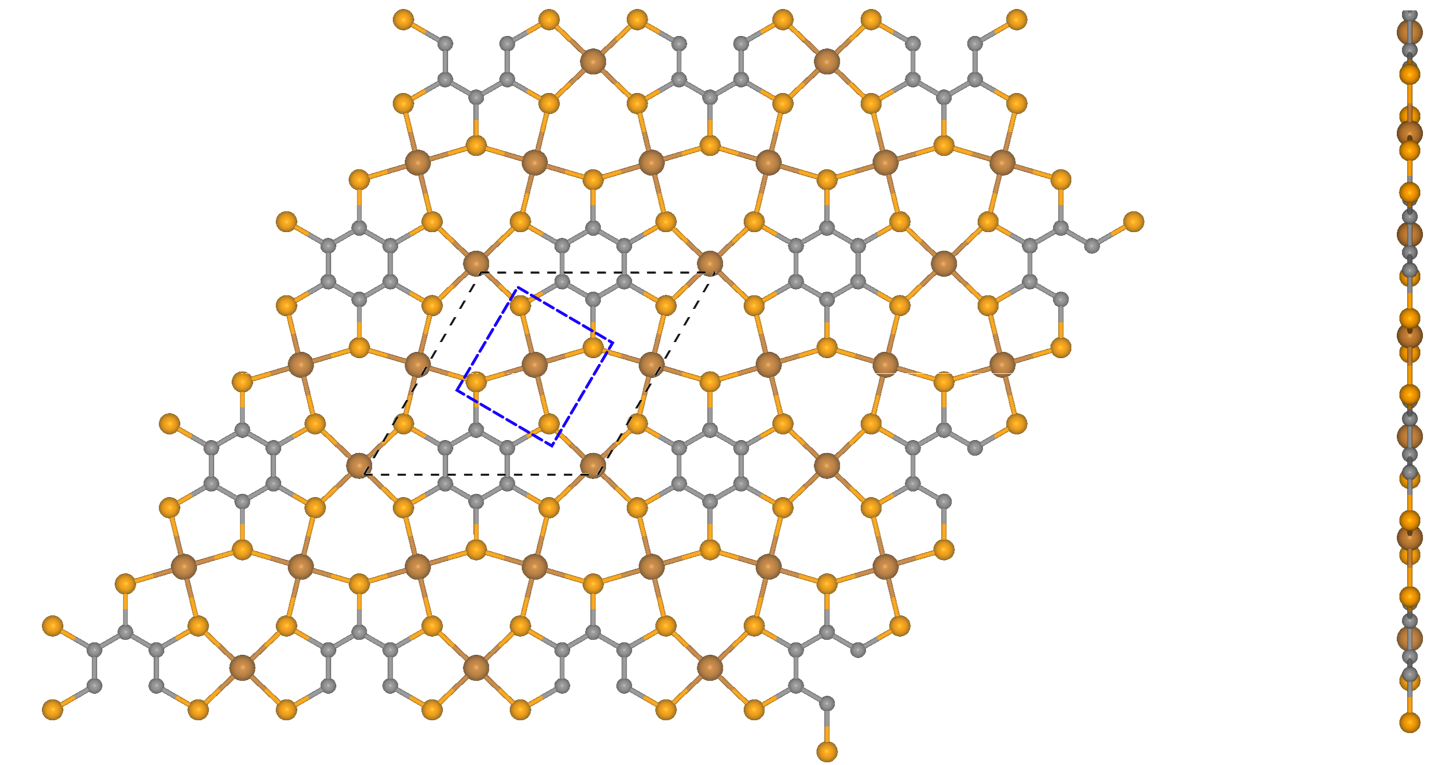


Figure S1. The unit cell of copper benzenehexaselenoate (Cu-BHS) a) top view and b) side view. The black dashed line denotes the unit cell. The blue dashed line denotes the CuX_4_ unit.


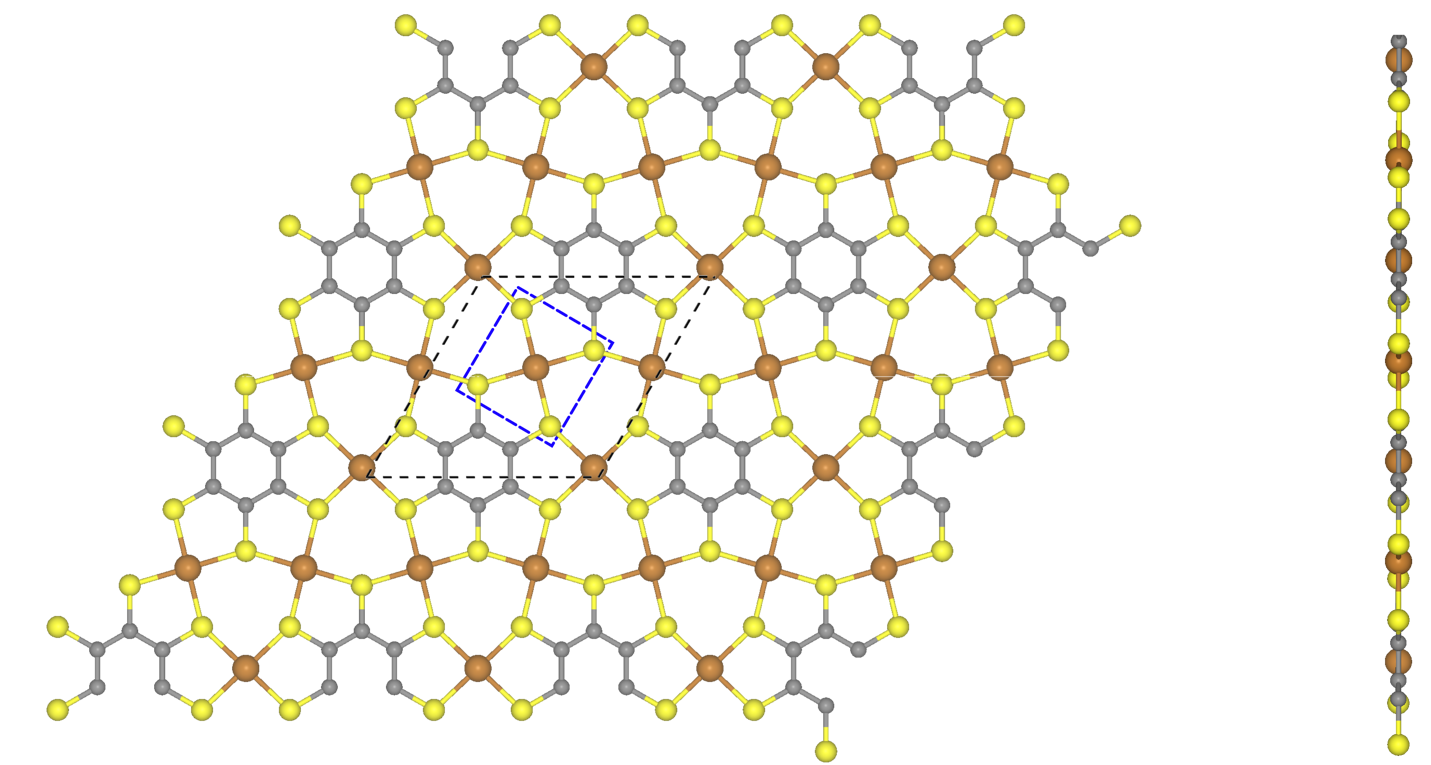


Figure S2. The unit cell of copper benzenehexathiol (Cu-BHT) a) top view and b) side view. The black dashed line denotes the unit cell. The blue dashed line denotes the CuX_4_ unit.


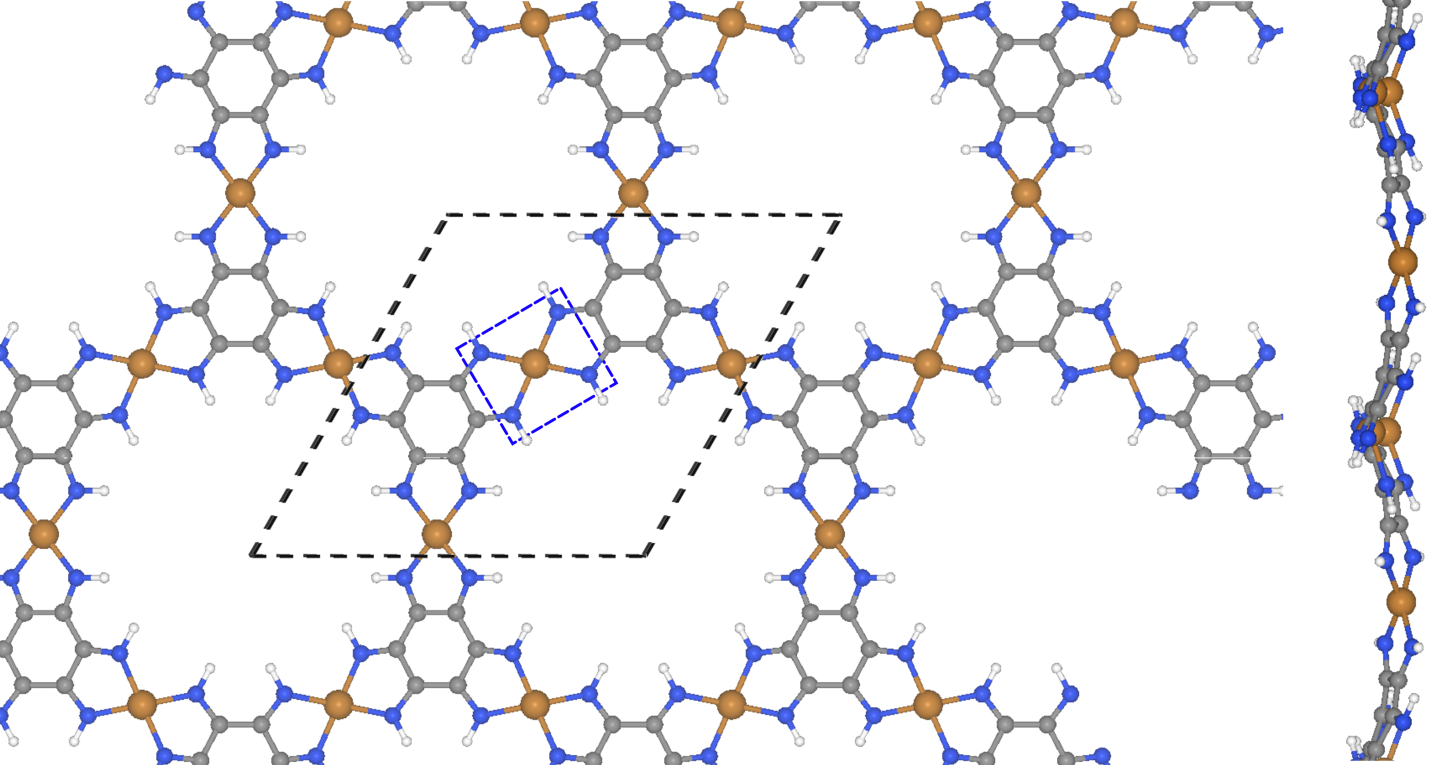


Figure S3. The unit cell of copper hexaiminobenzene (Cu-HIB) a) top view and b) side view. The black dashed line denotes the unit cell. The blue dashed line denotes the CuX4 unit.


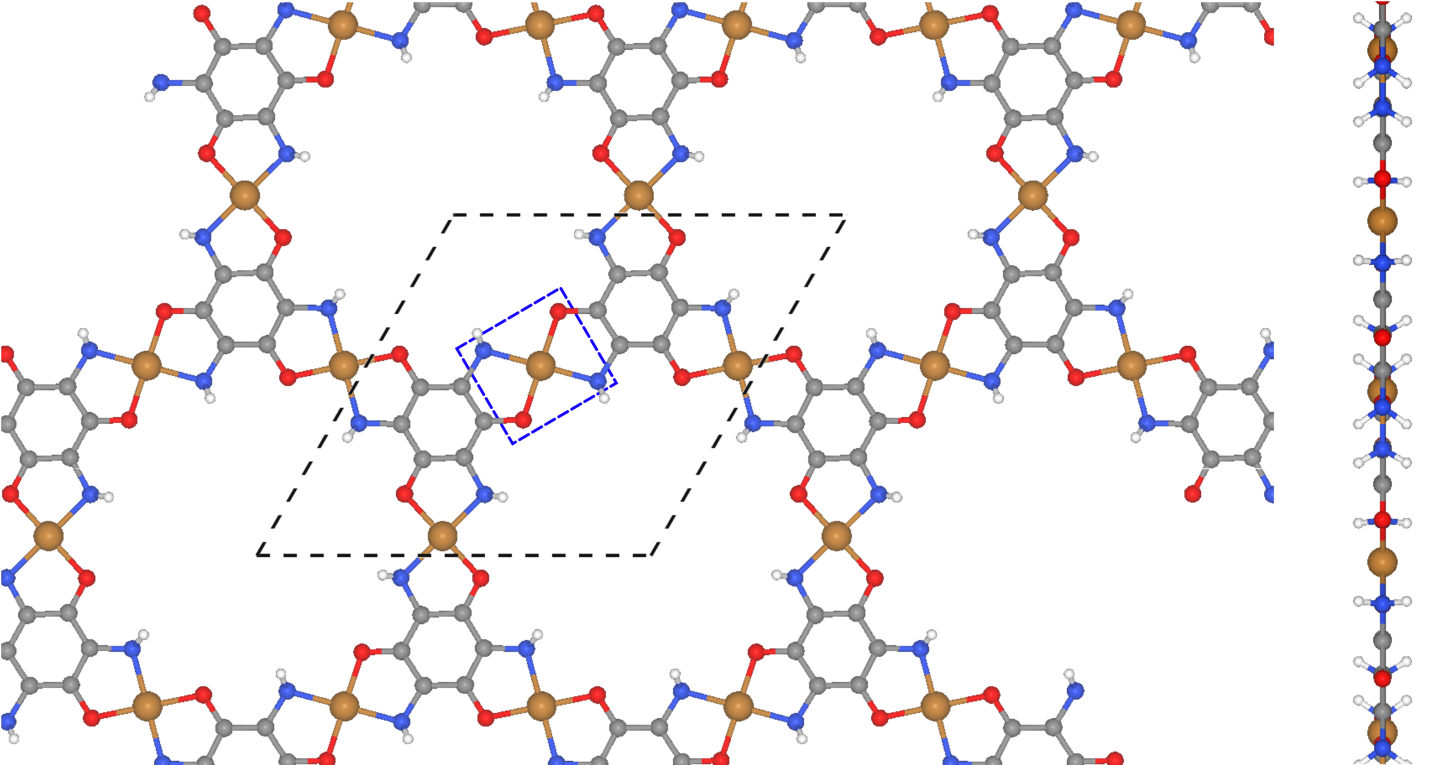


Figure S4. The unit cell of copper 1,3,5-triamino-2,4,6-benzenetriol (Cu-TABTO) a) top view and b) side view. The black dashed line denotes the unit cell. The blue dashed line denotes the CuX_2_Y_2_ unit.


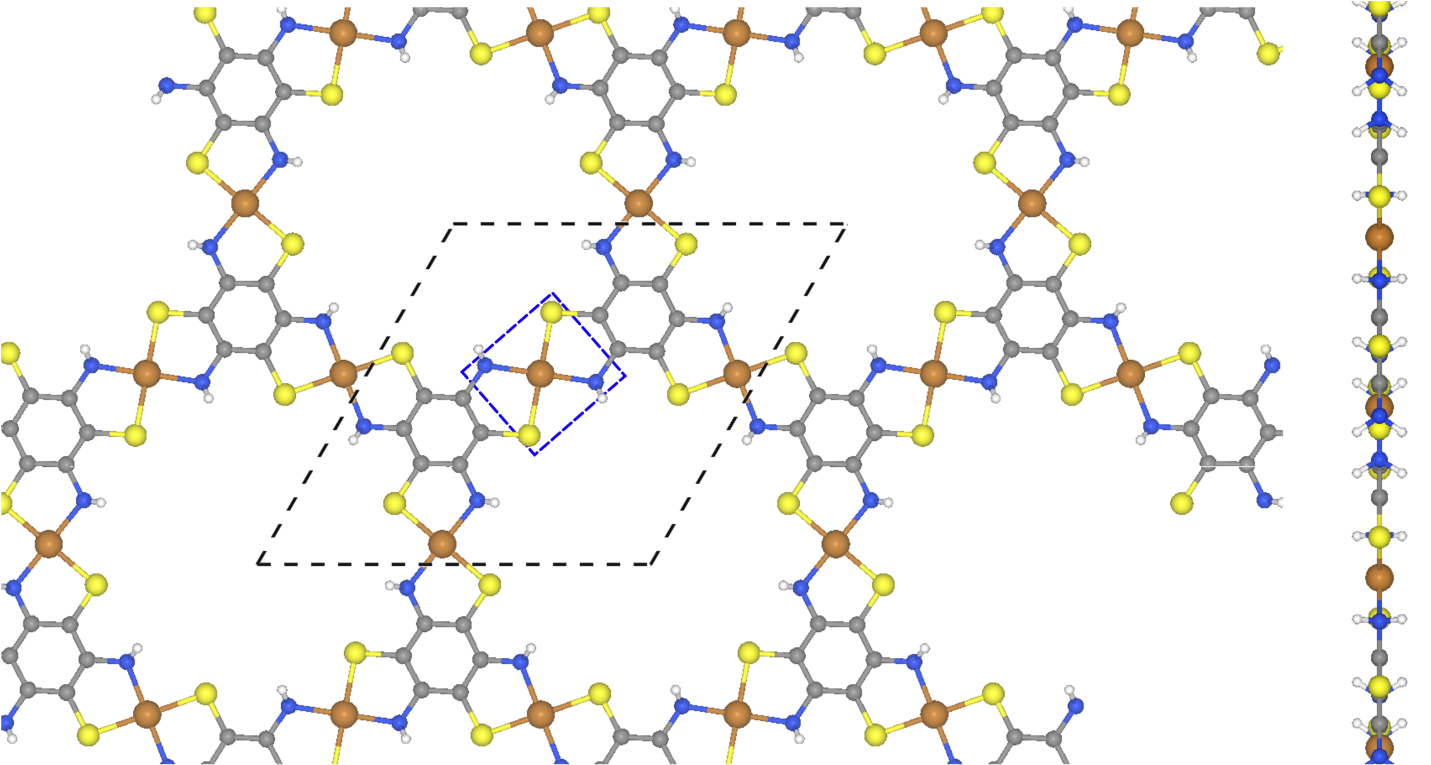


Figure S5. The unit cell of copper 1,3,5-triamino-2,4,6-benzenetrithiol (Cu-TABTT) a) top view and b) side view. The black dashed line denotes the unit cell. The blue dashed line denotes the CuX_2_Y_2_ unit.


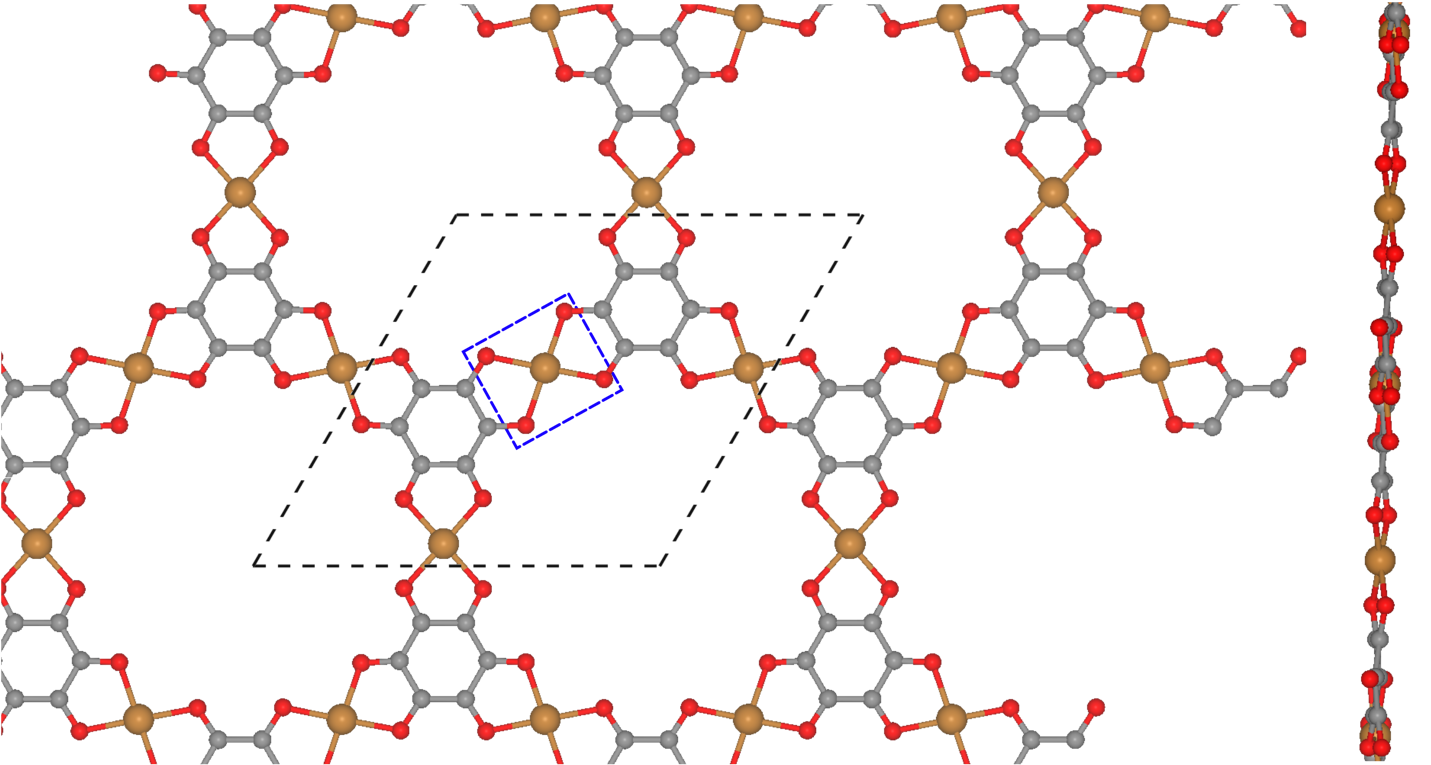


Figure S6. The unit cell of Cu-THQ a) top view and b) side view. The black dashed line denotes the unit cell. The blue dashed line denotes the CuX_4_ unit.


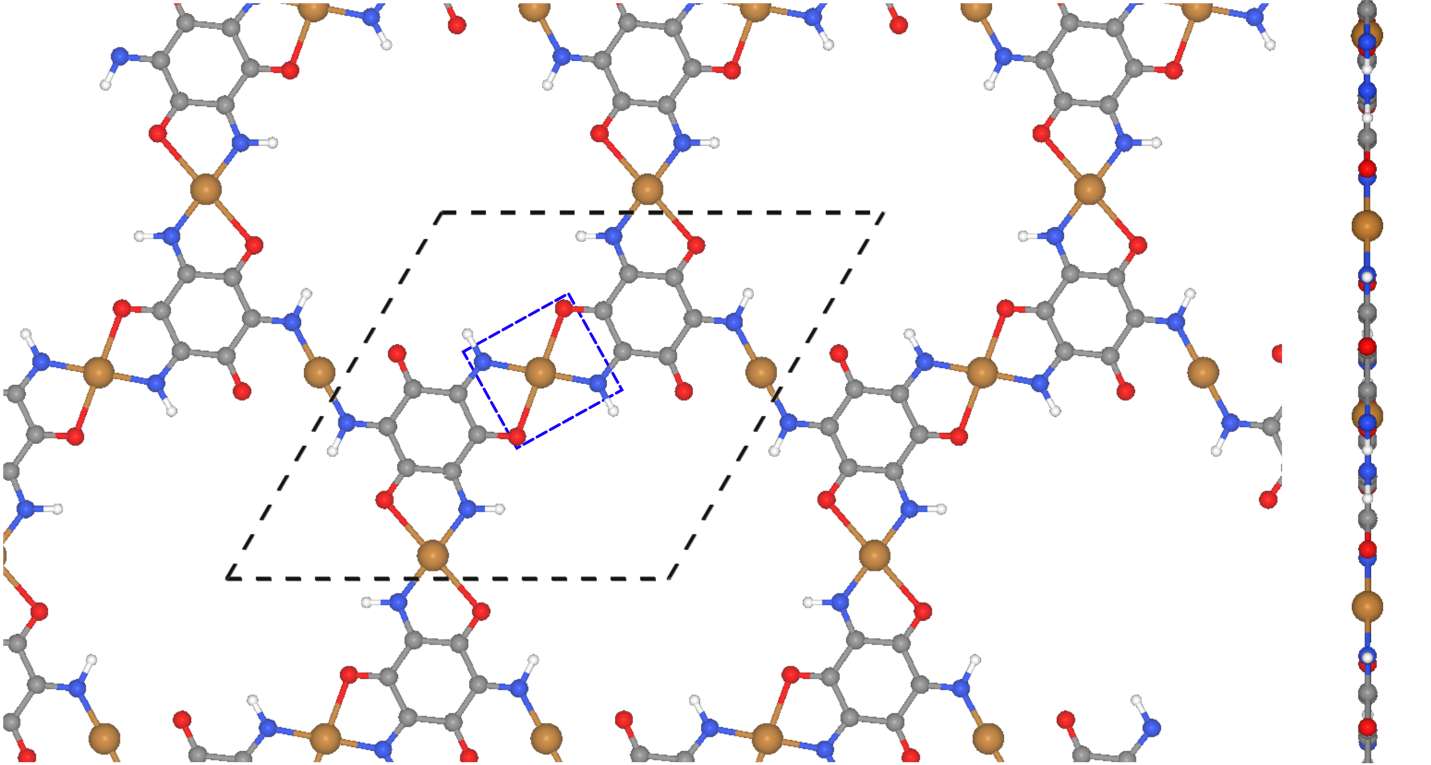


Figure S7. The unit cell of copper 1,3,5-triimino-2,4,6-benzenetriol (Cu-TIBTO) a) top view and b) side view. The black dashed line denotes the unit cell. The blue dashed line denotes the CuX_2_Y_2_ unit.


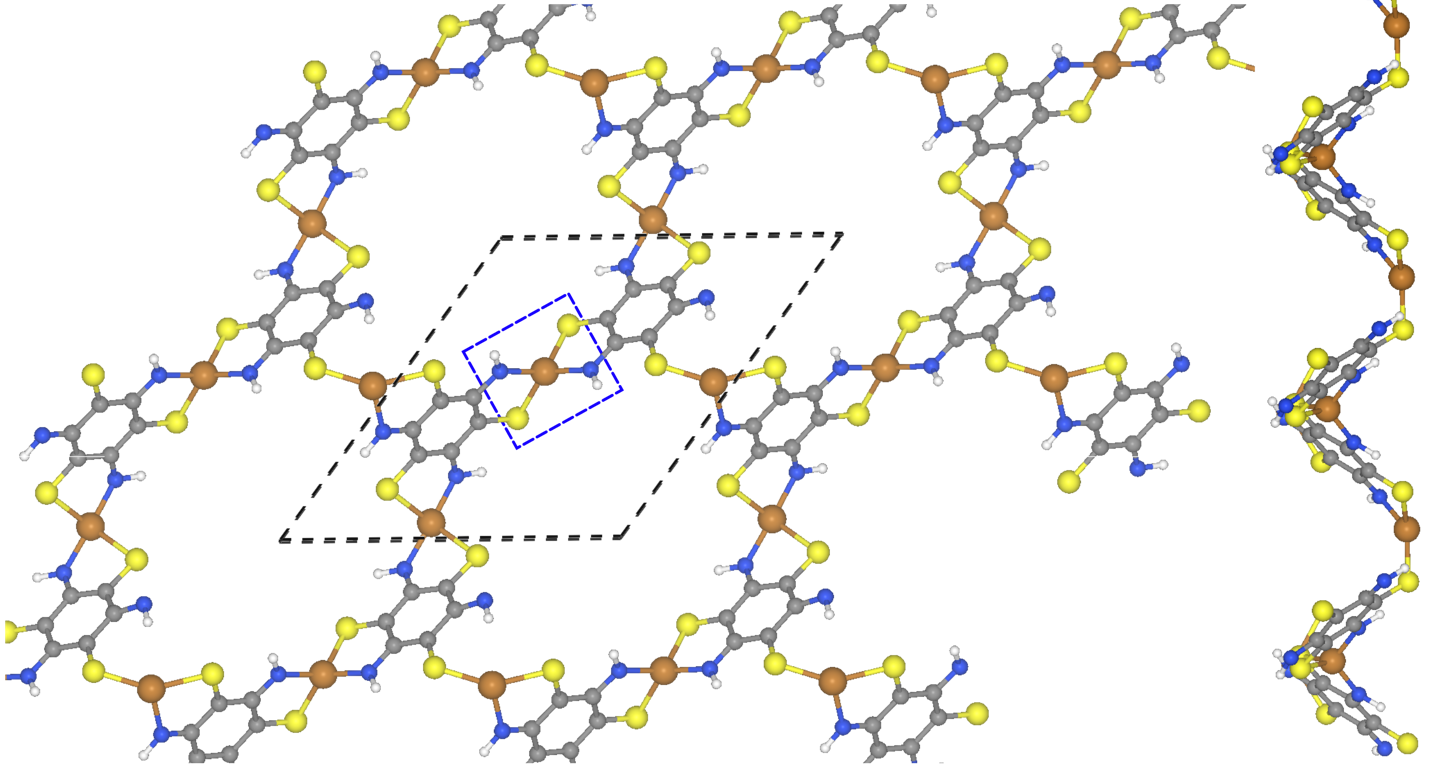


Figure S8. The unit cell of copper 1,3,5-triimino-2,4,6-benzenetrithiol (Cu-TIBTT) a) top view and b) side view. The black dashed line denotes the unit cell. The blue dashed line denotes the CuX_2_Y_2_ unit. Note that the copper atom adopts more of a tetrahedral coordination geometry in contrast to the square planar structure exhibited by the seven other Cu MOFs.

Table S2. DFT-calculated and experimental lattice parameters.

| **Cu MOF** | $\boldsymbol{a}_{\mathbf{DFT}}$ **(Å)** | $\boldsymbol{a}_{\mathbf{exp}}$ **(Å)** | $\boldsymbol{b}_{\mathbf{DFT}}$ **(Å)** | $\boldsymbol{b}_{\mathbf{exp}}$ **(Å)** | $\boldsymbol{\gamma}_{\mathbf{DFT}}$ **(^o^)** | $\boldsymbol{\gamma}_{\mathbf{exp}}$ **(^o^)** | **Ref.** |
| --- | --- | --- | --- | --- | --- | --- | --- |
| Cu-BHS | 9.199 | 15.323^a^ | 9.199 | 8.968^a^ | 60.000 | 90^a^ | ^[48]^ |
| Cu-BHT | 8.762 | 8.45 | 8.761 | 8.45 | 60.004 | n/a | ^[46,49]^ |
| Cu-HIB | 13.506 | 13.5(2) | 13.542 | 13.5(2) | 60.095 | 60 | ^[39–41,50]^ |
| Cu-TABTO | 13.441 | 13.41 | 13.423 | 13.41 | 60.083 | 60 | ^[42]^ |
| Cu-TABTT | 14.300 | 14.05^b^ | 14.304 | 14.05^b^ | 60.076 | 60^b^ | ^[43]^ |
| Cu-THQ | 13.413 | 13.108^c^ | 13.416 | 21.592^c^ | 60.010 | 90^c^ | ^[9,44]^ |
| Cu-TIBTO | 14.266 | n/a^d^ | 13.725 | n/a^d^ | 59.630 | n/a^d^ | ^[42]^ |
| Cu-TIBTT | 12.179 | n/a^d^ | 13.425 | n/a^d^ | 53.190 | n/a^d^ | ^[43]^ |

^a^ reported for slipping AA stacking

^b^ reported for isostructural Ni-TABTT

^c^ reported for AB slipped parallel stacking; also calculated to be 13.32 Å by Majidi et al.^[9]^ (Γ-point only)

^d^ not yet synthesized

Table S3. Structural parameters for the SBU in Cu-MOF structures.

| Structure | Metal | Linker 1 | Metal-Linker 1 Bond Length (Å) | Linker 2 | Metal-Linker 2 Bond Length (Å) | Linker-Linker Separation (Å) | Minimum Metal-Metal Separation (Å) | SBU Dihedral  (degrees) |
| --- | --- | --- | --- | --- | --- | --- | --- | --- |
| Cu-BHS | Cu | Se | 2.40 | Se | 2.40 | 3.31 | 4.60 | 0 |
| Cu-BHT | Cu | S | 2.28 | S | 2.29 | 3.15 | 4.38 | 0 |
| Cu-HIB | Cu | N | 1.97 | N | 1.96 | 2.58 | 6.79 | 43 |
| Cu-TABTO | Cu | N | 2.02 | O | 1.93 | 2.72 | 6.71 | 0 |
| Cu-TABTT | Cu | N | 2.03 | S | 2.28 | 3.00 | 7.15 | 0 |
| Cu-THQ | Cu | O | 1.99 | O | 1.99 | 2.64 | 6.71 | 19 |
| Cu-TIBTO | Cu | N | 1.89 | O | 2.20 | 2.66 | 6.86 | 0 |
| Cu-TIBTT | Cu | N | 1.93 | S | 2.27 | 2.89 | 6.68 | 78 |


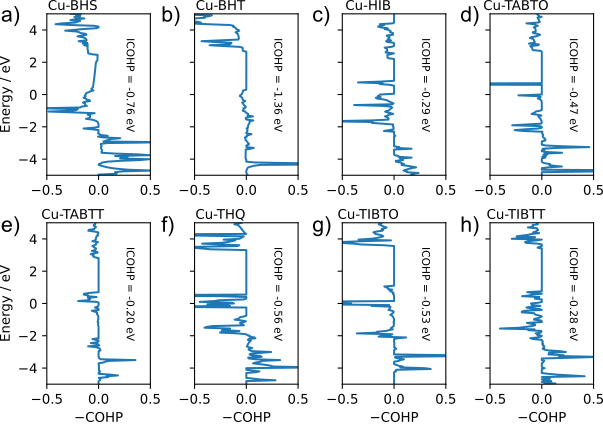


Figure S9: Crystal orbital Hamilton population (COHP) analysis for a) Cu-BHS, b) Cu-BHT, c) Cu-HIB, d) Cu-TABTO, e) Cu-TABTT, f) Cu-THQ, g) Cu-TIBTO, and h) Cu-TIBTT. Energies are relative to the Fermi energy. COHPs are integrated up to the Fermi energy and displayed in each subfigure.

DFT-calculated Energies

Table S4. DFT-calculated energies for clean Cu MOF surfaces.

| **Cu MOF** | **DFT Energy (E_DFT_) / eV** | **Gibbs Free Energy (G) / eV** |
| --- | --- | --- |
| Cu-BHS | -84.89 | -84.89 |
| Cu-BHT | -90.94 | -90.94 |
| Cu-HIB | -261.33 | -261.33 |
| Cu-TABTO | -252.68 | -252.68 |
| Cu-TABTT | -238.67 | -238.67 |
| Cu-THQ | -195.73 | -195.73 |
| Cu-TIBTO | -229.94 | -229.94 |
| Cu-TIBTT | -213.44 | -213.44 |

Table S5. DFT-calculated energies for gas-phase molecules.

| **Molecule** | **DFT Energy**  **(E_DFT_) / eV** | **Zero-Point Energy**  **(E_ZPE_) / eV** | **Thermal Correction**  **(–T∆S + ∫ c_p_ dT) / eV** | **Gibbs Free Energy**  **(G) / eV** |
| --- | --- | --- | --- | --- |
| CO | -14.78 | 0.13 | -0.47 | -15.12 |
| CO_2_ | -22.96 | 0.31 | -0.53 | -23.18 |
| H_2_ | -6.77 | 0.27 | -0.30 | -6.80 |
| H_2_O | -14.23 | 0.57 | -0.45 | -14.11 |
| HCOOH | -29.88 | 0.88 | -0.59 | -29.59 |

Table S6. DFT-calculated energies for *CO_2_ adsorption complexes.

| **Cu MOF** | **Site** | **Orientation** | **DFT Energy**  **(E_DFT_) / eV** | **Zero-Point Energy**  **(E_ZPE_) / eV** | **Thermal Correction**  **(–T∆S + ∫ c_v_ dT) / eV** | **Helmholtz Free Energy**  **(A) / eV** | **Adsorption Free Energy**  **(∆G) / eV** |
| --- | --- | --- | --- | --- | --- | --- | --- |
| Cu-BHS | on_Cu | parallel | -108.11 | 0.31 | -0.19 | -107.99 | 0.08 |
| Cu-BHT | on_Cu | parallel | -114.03 | 0.31 | -0.08 | -113.80 | 0.32 |
| Cu-HIB | on_Cu | parallel | -284.51 | 0.32 | -0.10 | -284.29 | 0.21 |
| Cu-TABTO | on_Cu | parallel | -275.82 | 0.24 | -0.09 | -275.67 | 0.19 |
| Cu-TABTT | on_Cu | parallel | -261.84 | 0.32 | -0.16 | -261.68 | 0.16 |
| Cu-THQ | on_Cu | parallel | -218.66 | 0.32 | -0.15 | -218.50 | 0.42 |
| Cu-TIBTO | on_Cu | parallel | -253.02 | 0.32 | -0.21 | -252.92 | 0.20 |
| Cu-TIBTT | on_Cu | parallel | -236.60 | 0.52 | -0.08 | -236.16 | 0.46 |

Table S7. DFT-calculated energies for *COOH adsorption complexes.

| **Cu MOF** | **Site** | **Orientation** | **DFT Energy**  **(E_DFT_) / eV** | **Zero-Point Energy**  **(E_ZPE_) / eV** | **Thermal Correction**  **(–T∆S + ∫ c_v_ dT) / eV** | **Gibbs Free Energy,**  **G / eV** | **Adsorption Free Energy,**  **∆G / eV** |
| --- | --- | --- | --- | --- | --- | --- | --- |
| Cu-BHS | on_Cu | parallel | -110.67 | 0.60 | -0.10 | -110.17 | 1.30 |
| Cu-BHS | on_Se_linker | parallel | -111.09 | 0.62 | -0.09 | -110.56 | 0.90 |
| Cu-BHT | on_Cu | parallel | -116.59 | 0.61 | -0.09 | -116.07 | 1.45 |
| Cu-BHT | on_S_linker | parallel | -117.10 | 0.62 | -0.09 | -116.56 | 0.96 |
| Cu-HIB | on_N_linker | parallel | -288.03 | 0.67 | -0.07 | -287.43 | 0.48 |
| Cu-HIB | on_Cu | parallel | -286.60 | 0.61 | -0.09 | -286.08 | 1.83 |
| Cu-TABTO | on_Cu | parallel | -278.71 | 0.62 | -0.09 | -278.17 | 1.09 |
| Cu-TABTO | on_N_linker | parallel | -278.09 | 0.52 | -0.10 | -277.66 | 1.60 |
| Cu-TABTO | on_O_linker | parallel | -278.69 | 0.67 | -0.08 | -278.10 | 1.16 |
| Cu-TABTT | on_Cu | parallel | -264.39 | 0.62 | -0.11 | -263.87 | 1.37 |
| Cu-TABTT | on_N_linker | parallel | -263.82 | 0.56 | -0.14 | -263.40 | 1.84 |
| Cu-TABTT | on_S_linker | parallel | -264.65 | 0.63 | -0.09 | -264.11 | 1.13 |
| Cu-THQ | on_Cu | parallel | -221.35 | 0.63 | -0.09 | -220.81 | 1.50 |
| Cu-THQ | on_O_linker | parallel | -221.74 | 0.67 | -0.07 | -221.14 | 1.17 |
| Cu-TIBTO | on_N_linker | parallel | -256.59 | 0.66 | -0.07 | -256.00 | 0.52 |
| Cu-TIBTO | on_O_linker | parallel | -256.09 | 0.66 | -0.09 | -255.52 | 1.00 |
| Cu-TIBTT | on_Cu | parallel | -237.98 | 0.56 | -0.13 | -237.55 | 2.47 |
| Cu-TIBTT | on_N_linker | parallel | -239.97 | 0.66 | -0.08 | -239.39 | 0.63 |
| Cu-TIBTT | on_S_linker | parallel | -239.50 | 0.64 | -0.09 | -238.96 | 1.06 |

Table S8. DFT-calculated energies for *OCHO adsorption complexes.

| **Cu MOF** | **Site** | **Orientation** | **DFT Energy**  **(E_DFT_) / eV** | **Zero-Point Energy**  **(E_ZPE_) / eV** | **Thermal Correction**  **(–T∆S + ∫ c_v_ dT) / eV** | **Gibbs Free Energy,**  **G / eV** | **Adsorption Free Energy,**  **∆G / eV** |
| --- | --- | --- | --- | --- | --- | --- | --- |
| Cu-BHS | on_Cu | parallel | -110.75 | 0.59 | -0.12 | -110.28 | 1.19 |
| Cu-BHS | on_Se_linker | parallel | -110.80 | 0.60 | -0.09 | -110.30 | 1.17 |
| Cu-BHT | on_Cu | parallel | -116.57 | 0.58 | -0.09 | -116.08 | 1.44 |
| Cu-BHT | on_S_linker | parallel | -116.61 | 0.60 | -0.09 | -116.11 | 1.41 |
| Cu-HIB | on_Cu | parallel | -287.29 | 0.58 | -0.12 | -286.82 | 1.08 |
| Cu-TABTO | on_Cu | parallel | -278.75 | 0.60 | -0.08 | -278.23 | 1.03 |
| Cu-TABTT | on_S_linker | parallel | -264.29 | 0.57 | -0.14 | -263.86 | 1.39 |
| Cu-THQ | on_Cu | parallel | -221.18 | 0.59 | -0.08 | -220.67 | 1.64 |
| Cu-TIBTO | on_Cu | parallel | -255.58 | 0.59 | -0.15 | -255.14 | 1.38 |
| Cu-TIBTT | on_Cu | parallel | -238.83 | 0.57 | -0.13 | -238.39 | 1.63 |
| Cu-TIBTT | on_N_linker | parallel | -238.58 | 0.56 | -0.14 | -238.15 | 1.87 |
| Cu-TIBTT | on_S_linker | parallel | -239.09 | 0.59 | -0.12 | -238.62 | 1.40 |

Table S9. DFT-calculated energies for *CO adsorption complexes.

| **Cu MOF** | **Site** | **Orientation** | **DFT Energy**  **(E_DFT_) / eV** | **Zero-Point Energy**  **(E_ZPE_) / eV** | **Thermal Correction**  **(–T∆S + ∫ c_v_ dT) / eV** | **Gibbs Free Energy,**  **G / eV** | **Adsorption Free Energy,**  **∆G / eV** |
| --- | --- | --- | --- | --- | --- | --- | --- |
| Cu-BHS | on_Cu | perpendicular | -100.35 | 0.18 | -0.08 | -100.25 | 0.50 |
| Cu-BHS | on_Se_linker | perpendicular | -99.96 | 0.14 | -0.16 | -99.97 | 0.79 |
| Cu-BHT | on_Cu | perpendicular | -106.12 | 0.18 | -0.11 | -106.05 | 0.76 |
| Cu-BHT | on_S_linker | perpendicular | -105.88 | 0.14 | -0.14 | -105.88 | 0.93 |
| Cu-HIB | on_Cu | perpendicular | -276.27 | 0.15 | -0.11 | -276.24 | 0.96 |
| Cu-HIB | on_N_linker | perpendicular | -276.27 | 0.15 | -0.18 | -276.30 | 0.90 |
| Cu-TABTO | on_Cu | perpendicular | -268.22 | 0.20 | -0.09 | -268.11 | 0.44 |
| Cu-TABTO | on_N_linker | perpendicular | -267.67 | 0.16 | -0.15 | -267.67 | 0.88 |
| Cu-TABTO | on_O_linker | perpendicular | -267.67 | 0.16 | -0.16 | -267.68 | 0.87 |
| Cu-TABTT | on_Cu | perpendicular | -253.59 | 0.18 | -0.02 | -253.44 | 1.10 |
| Cu-TABTT | on_N_linker | perpendicular | -253.61 | 0.15 | -0.08 | -253.54 | 1.00 |
| Cu-TABTT | on_S_linker | perpendicular | -253.64 | 0.15 | -0.10 | -253.59 | 0.95 |
| Cu-THQ | on_Cu | perpendicular | -211.02 | 0.19 | -0.13 | -210.95 | 0.65 |
| Cu-THQ | on_O_linker | perpendicular | -210.39 | 0.14 | -0.14 | -210.39 | 1.21 |
| Cu-TIBTO | on_Cu | perpendicular | -245.23 | 0.19 | -0.10 | -245.14 | 0.67 |
| Cu-TIBTO | on_N_linker | perpendicular | -245.24 | 0.19 | -0.11 | -245.16 | 0.65 |
| Cu-TIBTO | on_N_linker | perpendicular | -244.78 | 0.14 | -0.18 | -244.82 | 0.98 |
| Cu-TIBTO | on_O_linker | perpendicular | -244.76 | 0.14 | -0.13 | -244.76 | 1.05 |
| Cu-TIBTT | on_Cu | perpendicular | -228.36 | 0.14 | -0.22 | -228.44 | 0.87 |
| Cu-TIBTT | on_N_linker | perpendicular | -228.37 | 0.14 | -0.21 | -228.44 | 0.87 |
| Cu-TIBTT | on_S_linker | perpendicular | -228.36 | 0.14 | -0.16 | -228.37 | 0.94 |

Table S10. DFT-calculated energies for *H adsorption complexes.

| **Cu MOF** | **Site** | **DFT Energy**  **(E_DFT_) / eV** | **Zero-Point Energy**  **(E_ZPE_) / eV** | **Thermal Correction**  **(–T∆S + ∫ c_v_ dT) / eV** | **Gibbs Free Energy,**  **G / eV** | **Adsorption Free Energy,**  **∆G / eV** |
| --- | --- | --- | --- | --- | --- | --- |
| Cu-BHS | on_Cu | -87.29 | 0.11 | -0.06 | -87.23 | 1.06 |
| Cu-BHS | on_Se_linker | -88.18 | 0.21 | 0.00 | -87.97 | 0.32 |
| Cu-BHT | on_Cu | -93.20 | 0.13 | -0.02 | -93.08 | 1.26 |
| Cu-BHT | on_S_linker | -94.20 | 0.24 | 0.00 | -93.96 | 0.38 |
| Cu-HIB | on_Cu | -263.24 | 0.18 | 0.00 | -263.07 | 1.66 |
| Cu-HIB | on_N_linker | -264.89 | 0.33 | 0.00 | -264.56 | 0.17 |
| Cu-TABTO | on_Cu | -254.24 | 0.14 | -0.01 | -254.11 | 1.97 |
| Cu-TABTO | on_N_linker | -254.44 | 0.14 | -0.01 | -254.32 | 1.77 |
| Cu-TABTO | on_O_linker | -254.71 | 0.18 | 0.00 | -254.53 | 1.55 |
| Cu-TABTT | on_Cu | -241.67 | 0.22 | -0.01 | -241.45 | 0.61 |
| Cu-TABTT | on_N_linker | -241.68 | 0.22 | -0.01 | -241.46 | 0.61 |
| Cu-TABTT | on_S_linker | -241.63 | 0.23 | 0.00 | -241.40 | 0.67 |
| Cu-THQ | on_Cu | -197.52 | 0.14 | -0.01 | -197.39 | 1.74 |
| Cu-THQ | on_O_linker | -199.24 | 0.30 | 0.00 | -198.94 | 0.20 |
| Cu-TIBTO | on_Cu | -233.78 | 0.29 | 0.00 | -233.49 | -0.15 |
| Cu-TIBTO | on_N_linker | -233.62 | 0.33 | 0.00 | -233.30 | 0.04 |
| Cu-TIBTO | on_O_linker | -233.78 | 0.29 | 0.00 | -233.49 | -0.15 |
| Cu-TIBTT | on_Cu | -214.62 | 0.07 | -0.01 | -214.56 | 2.29 |
| Cu-TIBTT | on_N_linker | -217.36 | 0.33 | 0.00 | -217.03 | -0.19 |
| Cu-TIBTT | on_S_linker | -216.64 | 0.23 | 0.00 | -216.42 | 0.43 |


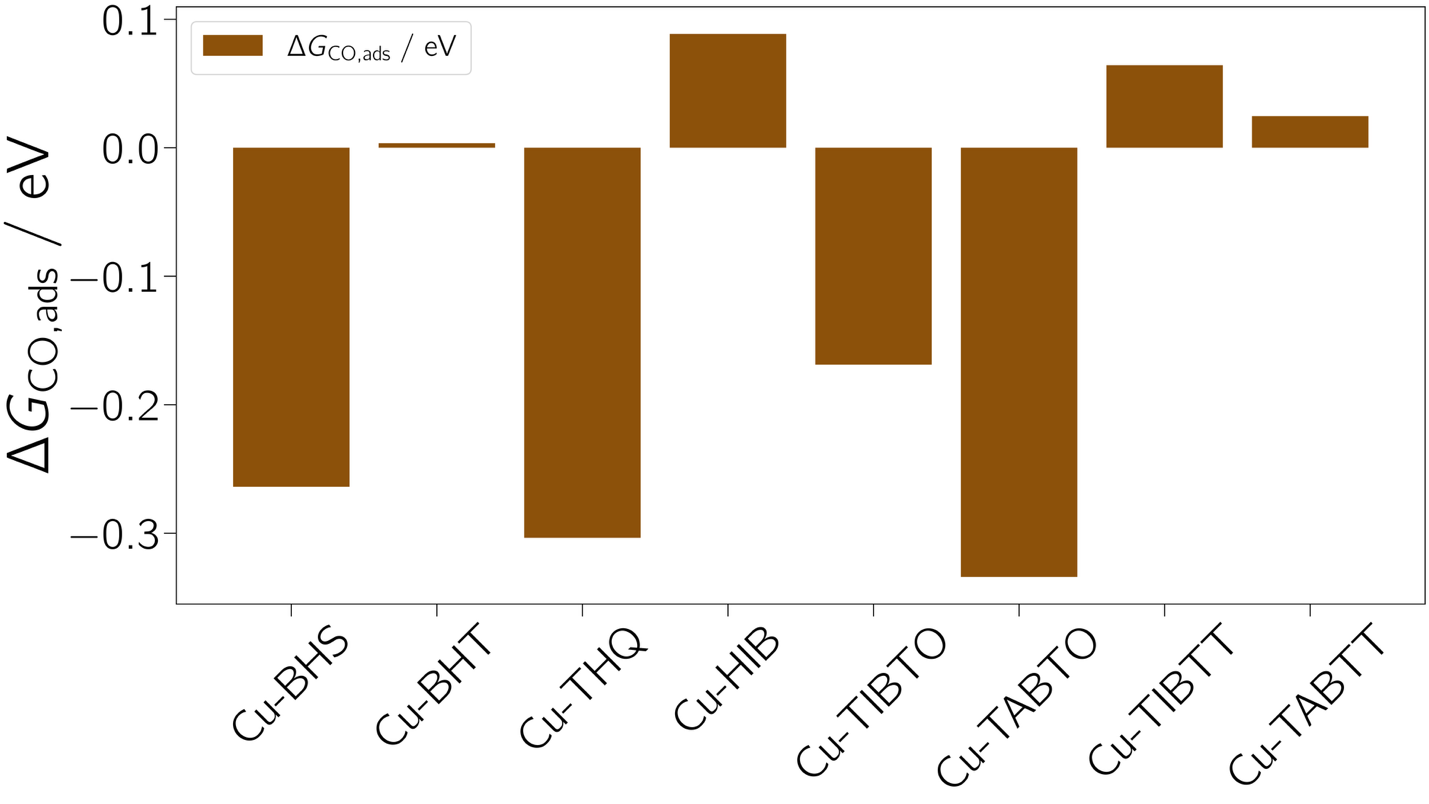


Figure S10. Gibbs free energies of adsorption for CO on Cu MOFs.


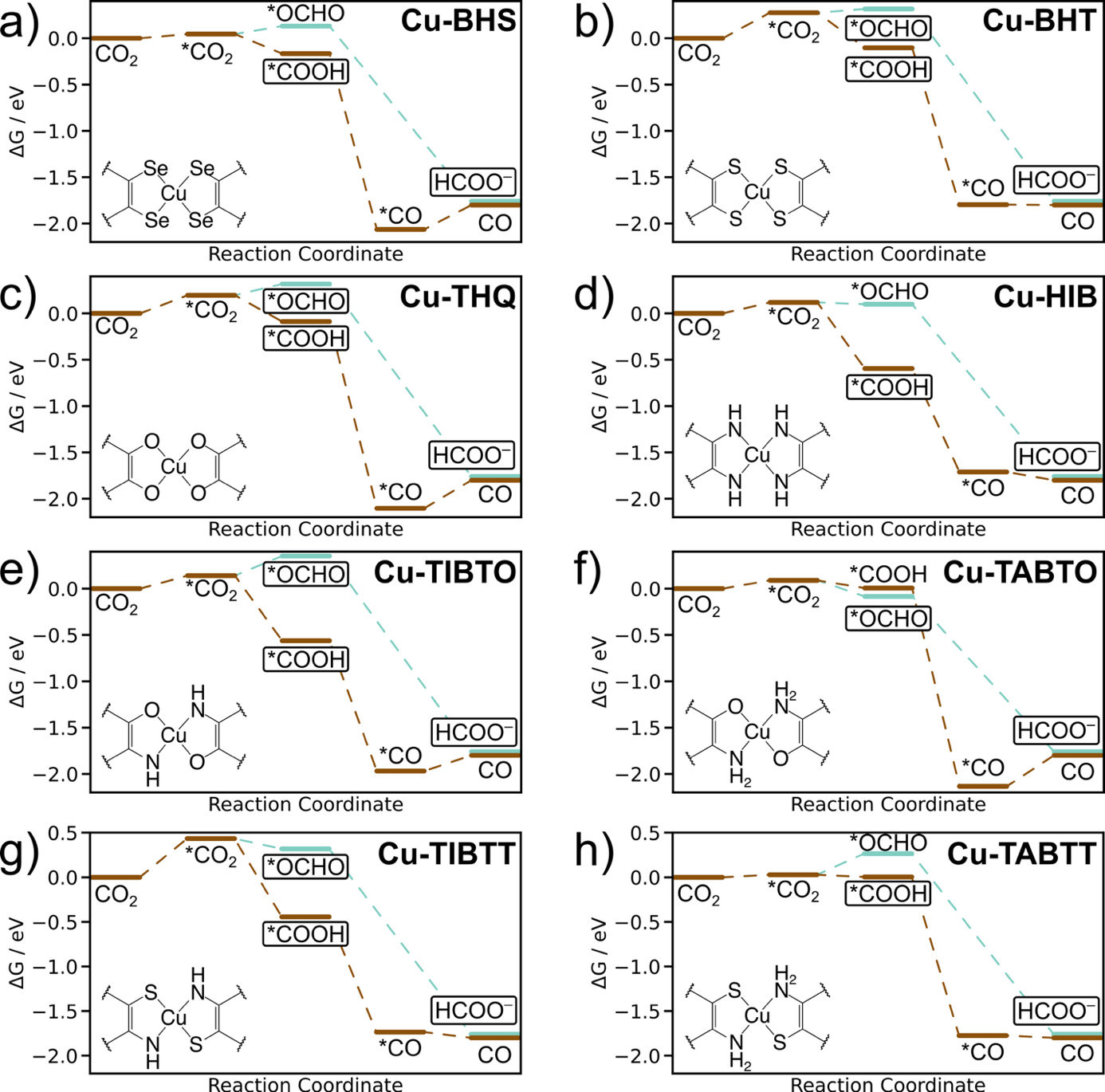


Figure S11: Free energy diagrams (at U = –1.0 V vs. RHE) for the MEP of CO_2_RR on the most stable sites of a) Cu-BHS, b) Cu-BHT, c) Cu-THQ, d) Cu-HIB, e) Cu-TIBTO, f) Cu-TABTO, g) Cu-TIBTT, and h) Cu-TABTT. The most stable sites are defined as the sites with the lowest adsorption free energy for a given intermediate.


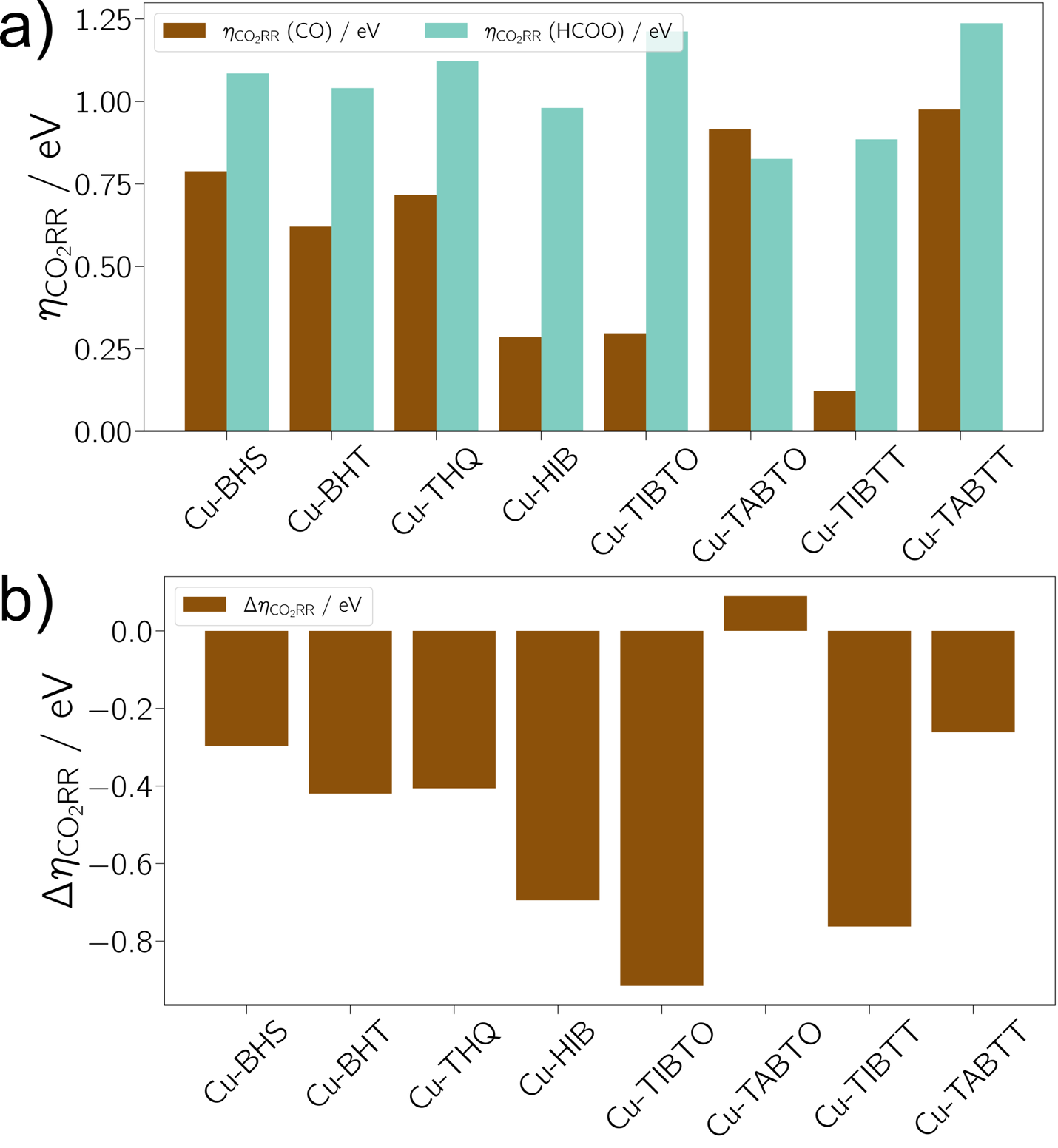


Figure S12. Theoretical overpotentials for CO_2_RR for CO and HCOO^–^ formation on Cu MOFs. a) The overpotentials for CO and HCOO^–^ formation are plotted separately. b) Overpotentials for CO and HCOO^–^ are plotted as the difference $\eta_{CO_{2}\mathrm{RR}}=\eta_{CO_{2}\mathrm{RR}}\left( \mathrm{CO} \right)-\eta_{CO_{2}\mathrm{RR}}\left( \mathrm{HCOO} \right)$.

**HER Free Energy Diagrams**


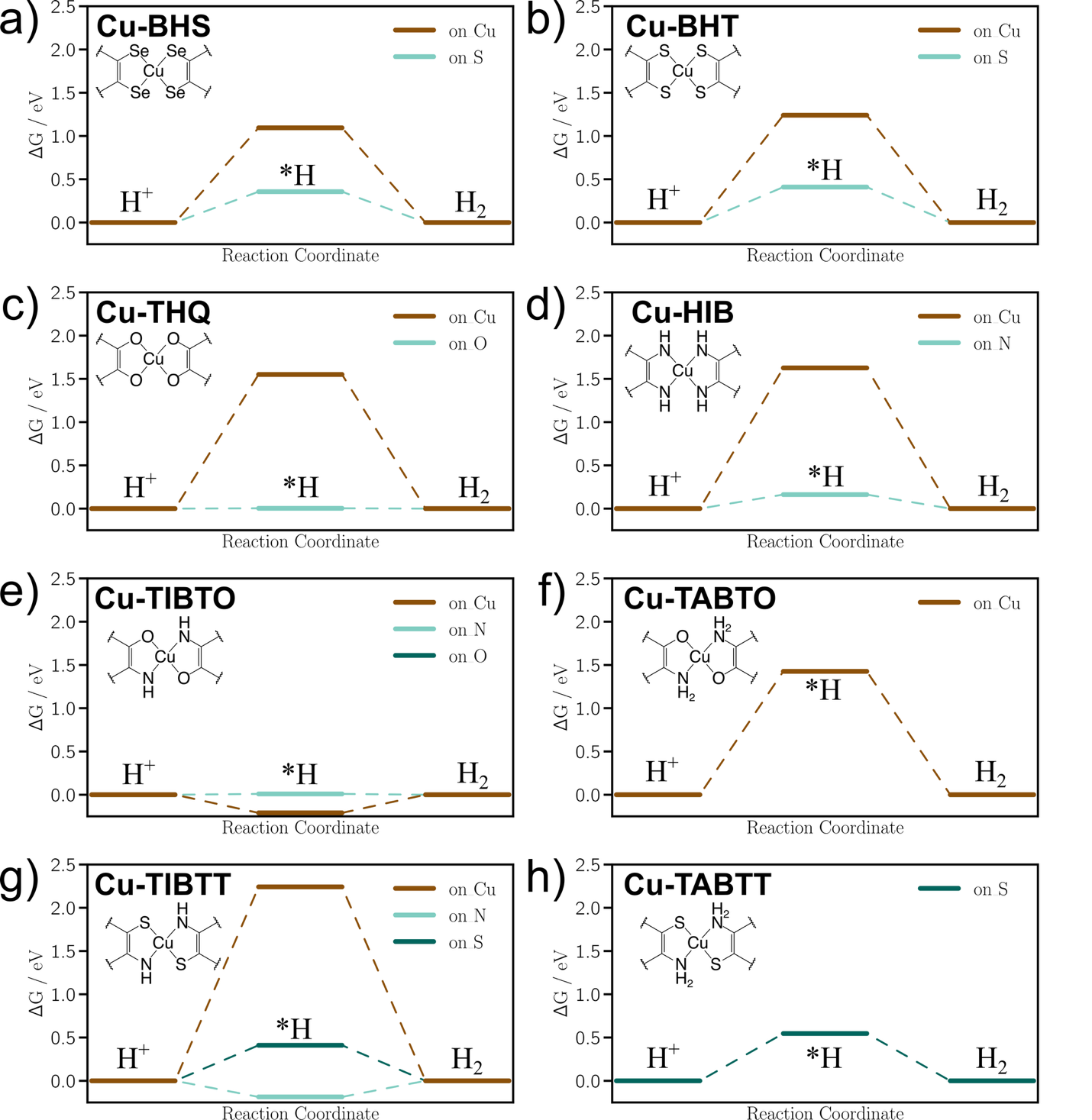


Figure S13. Free energy diagrams (at U = 0.0 V vs. RHE) for HER on the Cu and linker sites of a) Cu-BHS, b) Cu-BHT, c) Cu-THQ, d) Cu-HIB, e) Cu-TIBTO, f) Cu-TABTO, g) Cu-TIBTT, and h) Cu-TABTT.

**Activity Volcanos**


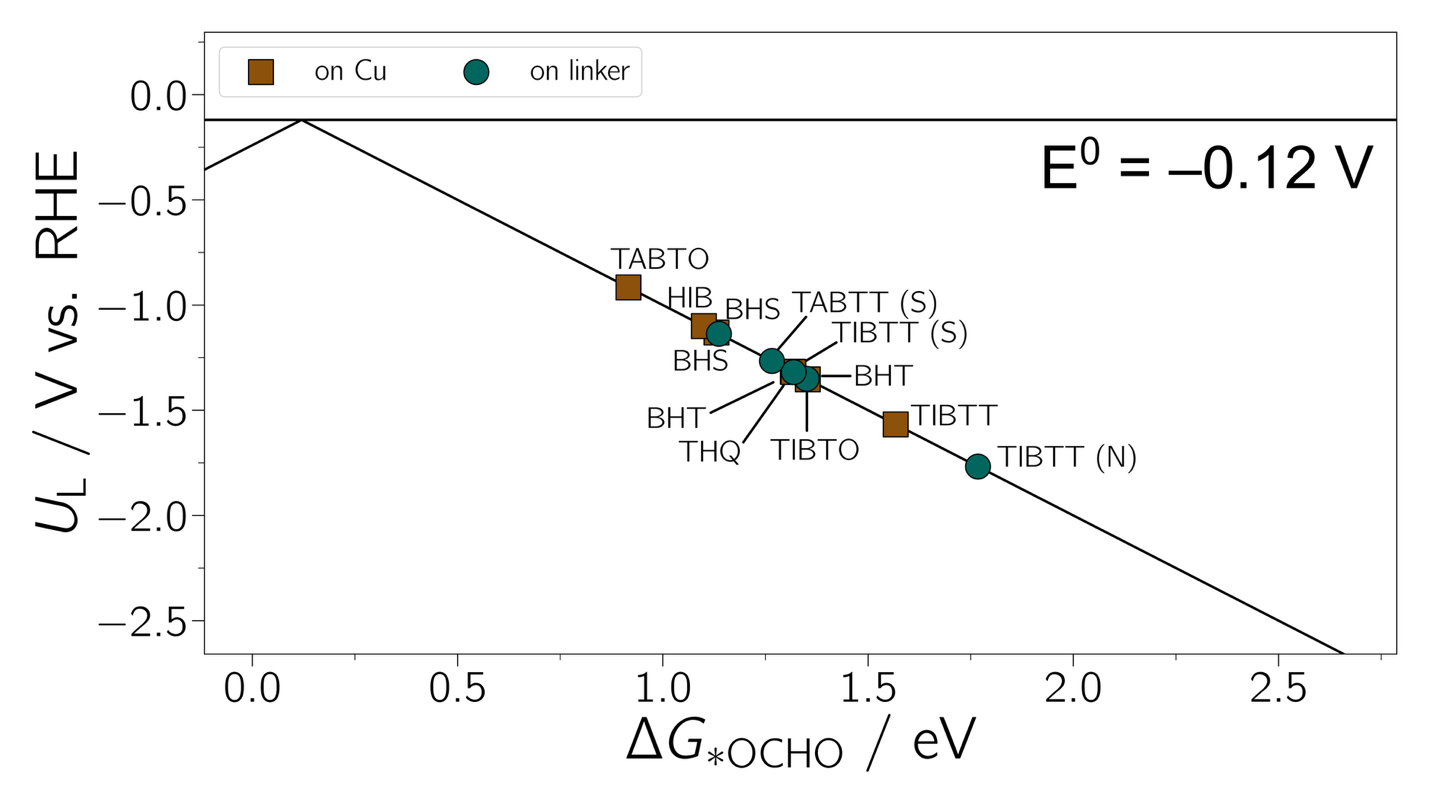


Figure S14. Activity volcano for HCOO^–^ production on Cu MOFs.

Table S11: Linker net atomic charges.

| Cu MOF | Linker Atom | Net Atomic Charge (\|e^–^\|) |
| --- | --- | --- |
| Cu-HIB | N | –0.56 |
| Cu-TIBTO | N | –0.53 |
| Cu-TIBTO | O | –0.41 |
| Cu-TIBTT | N | –0.50 |
| Cu-TIBTT | S | –0.18 |
| Cu-THQ | O | –0.34 |


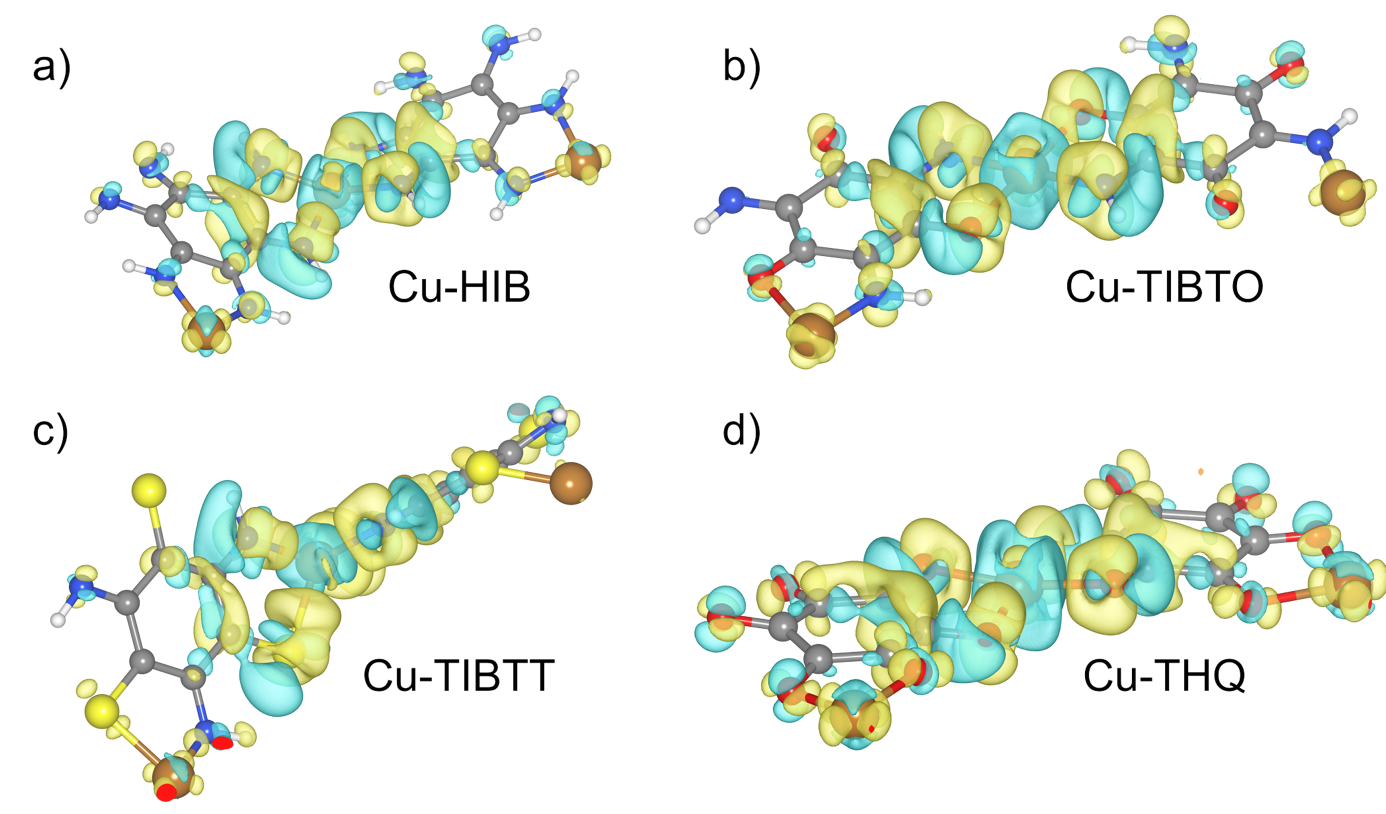


Figure S15. Valence charge density difference isosurfaces for a) Cu-HIB, b) Cu-TIBTO, c) Cu-TIBTT, and d) Cu-THQ. Isosurfaces are shown with an isovalue of 0.0005 Å^3^. Yellow and blue isosurfaces indicate regions of negative and positive charge accumulation, respectively.

Supplementary Note 3: Excluded Data Points for Cu MOFs in Figures 3 and 4

Note that certain Cu MOFs do not have reported energies for *OCHO complexes on all sites (**Table S7**). During structural relaxation, the OCHO intermediate migrates to the Cu site or abstracts a H atom from the Cu MOF to form desorbed HCOOH. Migration to Cu indicates that *OCHO is unstable on the linker atom relative to Cu. HCOOH formation suggests that the energies of the *OCHO intermediate and free formic acid are relatively close. In this case, the equilibrium potential for HCOO^–^ formation (Equation 5 to 7 in the main text) determines a lower bound for the *OCHO formation energy. This follows from the fact that the lack of a local minima corresponding to *OCHO suggests that HCOOH formation is favourable with respect to *OCHO. That the increased stability of singly reduced species on linker atoms does not extend to *OCHO may stem from the fact that *OCHO binds differently than *COOH. *OCHO binds to the catalyst surface via its oxygen atom(s) whereas *COOH typically binds solely via its carbon atom.

Supplementary Note 4: *OCHO on Cu-BHS and Cu-BHT

As shown in **Figure 1**, the Cu–Cu distances in Cu-BHS and Cu-BHT are significantly less than in other Cu MOFs. As such, bidentate adsorption on two Cu atoms is possible even while adsorbing over a linker atom. This may promote *OCHO adsorption on the linker atom for both Cu-BHS and Cu-BHT and enhance HCOO^–^ production. Additionally, we note that *OCHO is physiosorbed on Cu-TABTT. Favorable H-bonding interactions appear to stabilize the intermediate.

References

[1] J. Albo, D. Vallejo, G. Beobide, O. Castillo, P. Castaño, A. Irabien, *ChemSusChem* **2017**, *10*, 1100.

[2] J. Albo, M. Perfecto-Irigaray, G. Beobide, A. Irabien, *J. CO2 Util.* **2019**, *33*, 157.

[3] R. Chen, L. Cheng, J. Liu, Y. Wang, W. Ge, C. Xiao, H. Jiang, Y. Li, C. Li, *Small* **2022**, 2200720.

[4] L. Z. Dong, Y. F. Lu, R. Wang, J. Zhou, Y. Zhang, L. Zhang, J. Liu, S. L. Li, Y. Q. Lan, *Nano Res.* **2022**, *15*, 10185.

[5] R. Hinogami, S. Yotsuhashi, M. Deguchi, Y. Zenitani, H. Hashiba, Y. Yamada, *ECS Electrochem. Lett.* **2012**, *1*, H17.

[6] J. Liu, D. Yang, Y. Zhou, G. Zhang, G. Xing, Y. Liu, Y. Ma, O. Terasaki, S. Yang, L. Chen, *Angew. Chem. Int. Ed.* **2021**, *60*, 14473.

[7] Y. Liu, S. Li, L. Dai, J. Li, J. Lv, Z. Zhu, A. Yin, P. Li, B. Wang, *Angew. Chem. Int. Ed.* **2021**, *60*, 16409.

[8] Y.-Y. Liu, H.-L. Zhu, Z.-H. Zhao, N.-Y. Huang, P.-Q. Liao, X.-M. Chen, *ACS Catal.* **2022**, *12*, 2749.

[9] L. Majidi, A. Ahmadiparidari, N. Shan, S. N. Misal, K. Kumar, Z. Huang, S. Rastegar, Z. Hemmat, X. Zou, P. Zapol, J. Cabana, L. A. Curtiss, A. Salehi-Khojin, L. Majidi, A. Ahmadiparidari, S. N. Misal, S. Rastegar, Z. Hemmat, A. Salehi-Khojin, N. Shan, P. Zapol, L. A. Curtiss, K. Kumar, J. Cabana, Z. Huang, X. Zou, *Adv. Mater.* **2021**, *33*, 2004393.

[10] Z. Meng, J. Luo, W. Li, K. A. Mirica, *J. Am. Chem. Soc.* **2020**, *142*, 21656.

[11] D. H. Nam, O. S. Bushuyev, J. Li, P. De Luna, A. Seifitokaldani, C. T. Dinh, F. P. García De Arquer, Y. Wang, Z. Liang, A. H. Proppe, C. S. Tan, P. Todorović, O. Shekhah, C. M. Gabardo, J. W. Jo, J. Choi, M. J. Choi, S. W. Baek, J. Kim, D. Sinton, S. O. Kelley, M. Eddaoudi, E. H. Sargent, *J. Am. Chem. Soc.* **2018**, *140*, 11378.

[12] X. F. Qiu, H. L. Zhu, J. R. Huang, P. Q. Liao, X. M. Chen, *J. Am. Chem. Soc.* **2021**, *143*, 7242.

[13] R. Senthil Kumar, S. Senthil Kumar, M. Anbu Kulandainathan, *Electrochem. Commun.* **2012**, *25*, 70.

[14] X. Tan, C. Yu, C. Zhao, H. Huang, X. Yao, X. Han, W. Guo, S. Cui, H. Huang, J. Qiu, *ACS Appl. Mater. Interfaces* **2019**, *11*, 9904.

[15] T. Van Phuc, J. S. Chung, S. H. Hur, *Catalysts* **2021**, *11*, 537.

[16] T. Van Phuc, S. G. Kang, J. S. Chung, S. H. Hur, *Mater. Res. Bull.* **2021**, *138*, 111228.

[17] L. Wang, X. Li, L. Hao, S. Hong, A. W. Robertson, Z. Sun, *Chin. J. Catal.* **2022**, *43*, 1049.

[18] S. Wei, X. Jiang, C. He, S. Wang, Q. Hu, X. Chai, X. Ren, H. Yang, C. He, *J. Mater. Chem. A* **2022**, *10*, 6187.

[19] J. X. Wu, S. Z. Hou, X. D. Zhang, M. Xu, H. F. Yang, P. S. Cao, Z. Y. Gu, *Chem. Sci.* **2019**, *10*, 2199.

[20] Y. H. Xiao, Y. X. Zhang, R. Zhai, Z. G. Gu, J. Zhang, *Sci. China Mater.* **2021**, *65*, 1269.

[21] T. Yan, P. Wang, Z.-H. Xu, W.-Y. Sun, *ACS Appl. Mater. Interfaces* **2022**, *14*, 13645.

[22] J. D. Yi, R. Xie, Z. L. Xie, G. L. Chai, T. F. Liu, R. P. Chen, Y. B. Huang, R. Cao, *Angew. Chem. Int. Ed.* **2020**, *59*, 23641.

[23] Y. Zhang, L. Z. Dong, S. Li, X. Huang, J. N. Chang, J. H. Wang, J. Zhou, S. L. Li, Y. Q. Lan, *Nat. Commun.* **2021**, *12*, 1.

[24] Z. H. Zhao, K. Zheng, N. Y. Huang, H. L. Zhu, J. R. Huang, P. Q. Liao, X. M. Chen, *Chem. Commun.* **2021**, *57*, 12764.

[25] Z. H. Zhao, H. L. Zhu, J. R. Huang, P. Q. Liao, X. M. Chen, *ACS Catal.* **2022**, *12*, 7986.

[26] H. Zhong, M. Ghorbani-Asl, K. H. Ly, J. Zhang, J. Ge, M. Wang, Z. Liao, D. Makarov, E. Zschech, E. Brunner, I. M. Weidinger, J. Zhang, A. V. Krasheninnikov, S. Kaskel, R. Dong, X. Feng, *Nat. Commun.* **2020**, *11*, 1.

[27] Y. Zhou, S. Chen, S. Xi, Z. Wang, P. Deng, F. Yang, Y. Han, Y. Pang, B. Y. Xia, *Cell Rep. Phys. Sci.* **2020**, *1*, 100182.

[28] X. Zhou, J. Dong, Y. Zhu, L. Liu, Y. Jiao, H. Li, Y. Han, K. Davey, Q. Xu, Y. Zheng, S. Z. Qiao, *J. Am. Chem. Soc.* **2021**, *143*, 6681.

[29] H. L. Zhu, J. R. Huang, X. W. Zhang, C. Wang, N. Y. Huang, P. Q. Liao, X. M. Chen, *ACS Catal.* **2021**, *11*, 11786.

[30] L. L. Zhuo, P. Chen, K. Zheng, X. W. Zhang, J. X. Wu, D. Y. Lin, S. Y. Liu, Z. S. Wang, J. Y. Liu, D. D. Zhou, J. P. Zhang, *Angew. Chem. Int. Ed.* **2022**, *61*, e202204967.

[31] X. Huang, H. Yao, Y. Cui, W. Hao, J. Zhu, W. Xu, D. Zhu, *ACS Applied Materials and Interfaces* **2017**, *9*, 40752.

[32] A. S. Duke, E. A. Dolgopolova, R. P. Galhenage, S. C. Ammal, A. Heyden, M. D. Smith, D. A. Chen, N. B. Shustova, *J. Phys. Chem. C* **2015**, *119*, 27457.

[33] W. Graham, T. Jenkins, C. Fivecoat, J. H. West, E. C. Hill, Y. Zeng, S. Ullah, T. Thonhauser, *J. Mater. Chem. A* **2025**, *13*, 30661.

[34] Y. Lv, S. Zhang, Z. Li, C. Jing, M. Shuai, W. Zhu, Z. Hu, J.-Q. Wang, L. Zhang, *Small* **2026**, *22*, e12635.

[35] S. Jia, Q. Zhu, X. Chen, C. Xue, M. Dong, T. Deng, H. Cheng, T. Yao, J. Jiao, Z. Xia, J. Zeng, C. Chen, H. Wu, M. He, B. Han, *J. Am. Chem. Soc.* **2025**, *147*, 22580.

[36] Y. Zhang, L. Zhang, X. Feng, G. Zhang, Y. Zhang, *Small* **2025**, *21*, e05892.

[37] G. Xing, L. Cheng, K. Li, Y. Gao, H. Tang, Y. Wang, Z. Wu, *New J. Chem.* **2020**, *44*, 12299.

[38] G. Xing, S. Liu, J. yao Liu, *International Journal of Hydrogen Energy* **2023**, *48*, 3486.

[39] M. Tang, H. Shen, Q. Sun, *J. Phys. Chem. C* **2019**, *123*, 26460.

[40] J. H. Dou, L. Sun, Y. Ge, W. Li, C. H. Hendon, J. Li, S. Gul, J. Yano, E. A. Stach, M. Dincǎ, *J. Am. Chem. Soc.* **2017**, *139*, 13608.

[41] D. Feng, T. Lei, M. R. Lukatskaya, J. Park, Z. Huang, M. Lee, L. Shaw, S. Chen, A. A. Yakovenko, A. Kulkarni, J. Xiao, K. Fredrickson, J. B. Tok, X. Zou, Y. Cui, Z. Bao, *Nat. Energy* **2018**, *3*, 30.

[42] Y. Jiang, I. Oh, S. H. Joo, Y. S. Seo, S. H. Lee, W. K. Seong, Y. J. Kim, J. Hwang, S. K. Kwak, J. W. Yoo, R. S. Ruoff, *J. Am. Chem. Soc.* **2020**, *142*, 18346.

[43] X. Sun, K. H. Wu, R. Sakamoto, T. Kusamoto, H. Maeda, X. Ni, W. Jiang, F. Liu, S. Sasaki, H. Masunaga, H. Nishihara, *Chem. Sci.* **2017**, *8*, 8078.

[44] J. Park, A. C. Hinckley, Z. Huang, D. Feng, A. A. Yakovenko, M. Lee, S. Chen, X. Zou, Z. Bao, *J. Am. Chem. Soc.* **2018**, *140*, 14533.

[45] L. Yang, S. Feng, W. Zhu, *Journal of Hazardous Materials* **2023**, *445*, 130534.

[46] X. Huang, P. Sheng, Z. Tu, F. Zhang, J. Wang, H. Geng, Y. Zou, C. A. Di, Y. Yi, Y. Sun, W. Xu, D. Zhu, *Nat. Commun.* **2015**, *6*, 1.

[47] X. Huang, S. Zhang, L. Liu, L. Yu, G. Chen, W. Xu, D. Zhu, *Angew. Chem. Int. Ed.* **2018**, *57*, 146.

[48] Y. Cui, J. Yan, Z. Chen, J. Zhang, Y. Zou, Y. Sun, W. Xu, D. Zhu, *Adv. Sci.* **2019**, *6*, 1802235.

[49] F. Li, X. Zhang, X. Liu, M. Zhao, *ACS Appl. Mater. Interfaces* **2018**, *10*, 15012.

[50] N. Lahiri, N. Lotfizadeh, R. Tsuchikawa, V. V. Deshpande, J. Louie, *J. Am. Chem. Soc.* **2017**, *139*, 19.
